# Supplementary material for: Long-Term Survival Outcomes of Cytoreductive Nephrectomy Combined with Targeted Therapy for Metastatic Renal Cell Carcinoma: A Systematic Review and Individual Patient Data Meta-Analysis
Source: Cancers (Basel). 2021 Feb 9;13(4):695. doi: 10.3390/cancers13040695 (PMC7915816; doi:10.3390/cancers13040695)
Supplement: Supplementary file 1 [file cancers-13-00695-s001.zip › Supplemental Data File 2.pdf]

Overall Survival

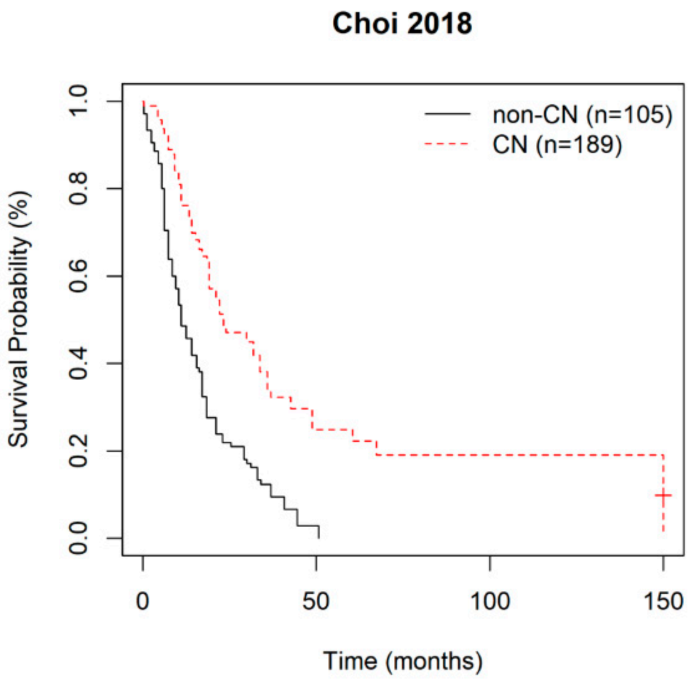

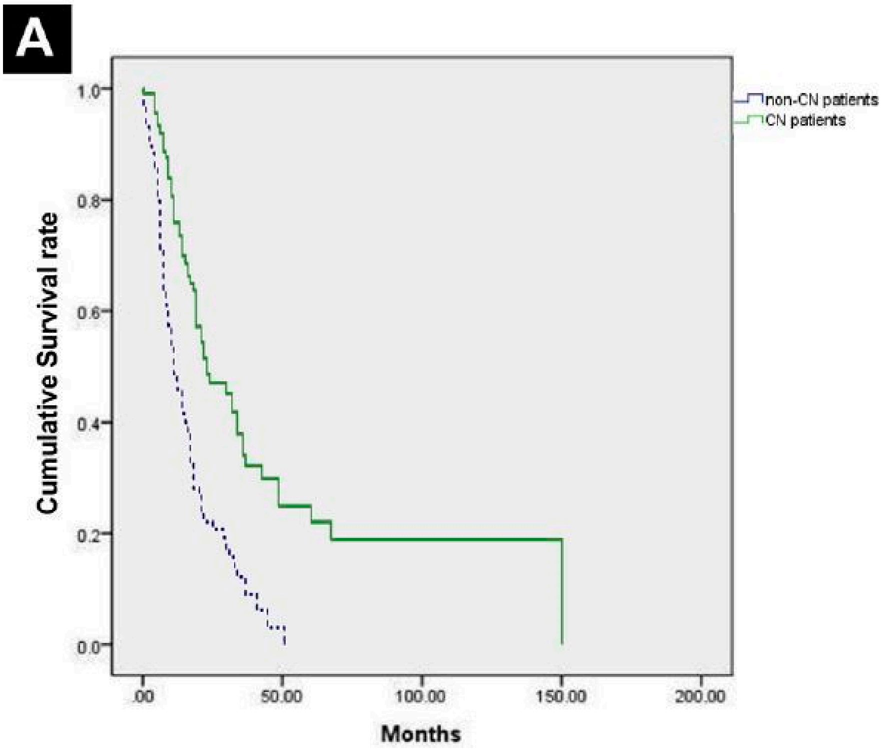

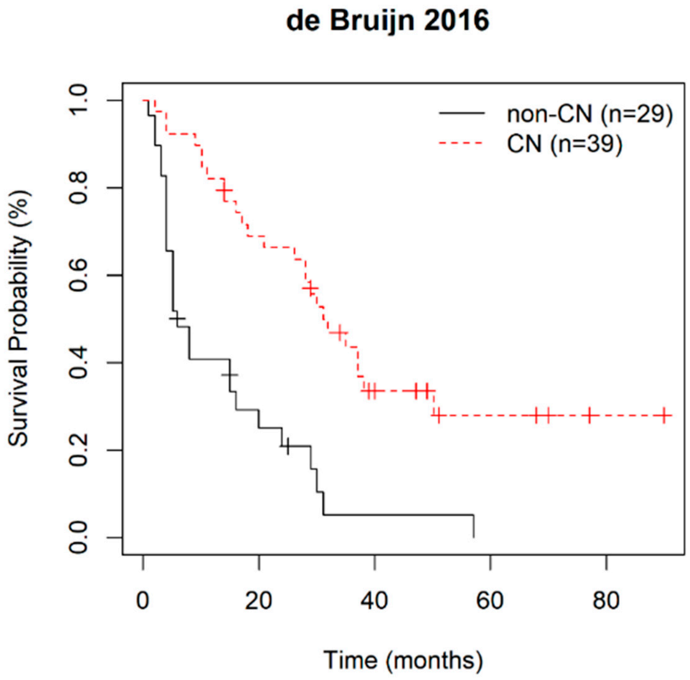
de Bruijn 2016
non-CN (n=29)
CN (n=39)
Survival Probability (%)
Time (months)
0.0
0.2
0.4
0.6
0.8
1.0
0
20
40
60
80

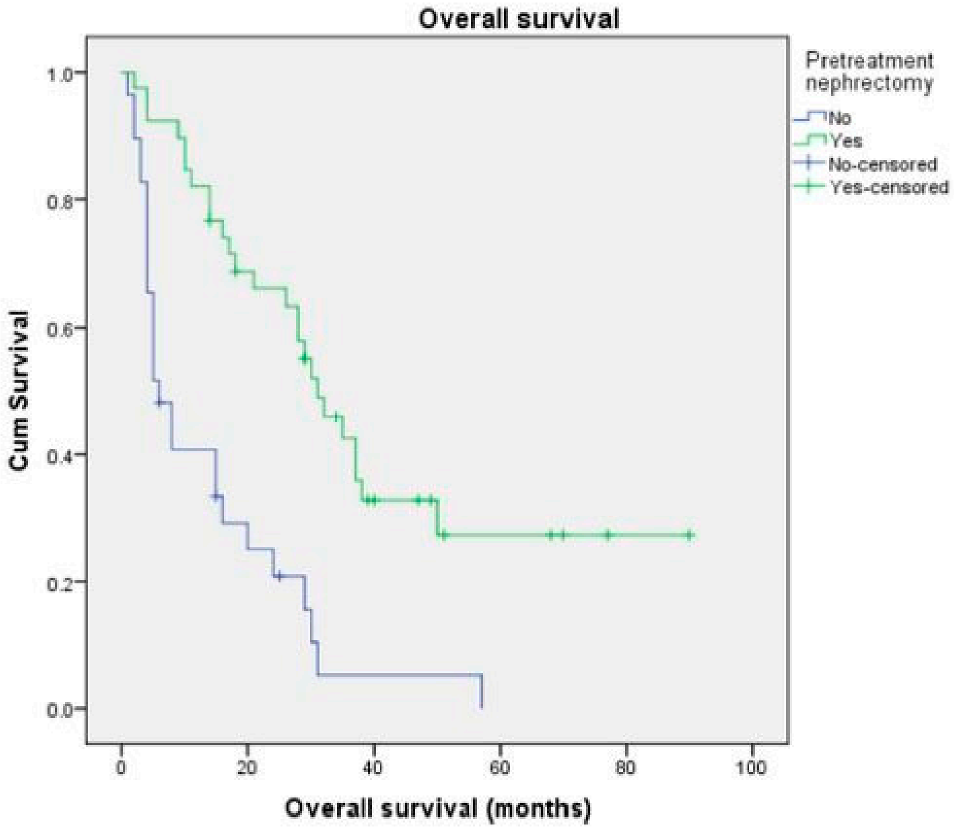
Overall survival
Pretreatment nephrectomy
No
Yes
No-censored
Yes-censored
Cum Survival
Overall survival (months)
0.0
0.2
0.4
0.6
0.8
1.0
0
20
40
60
80
100

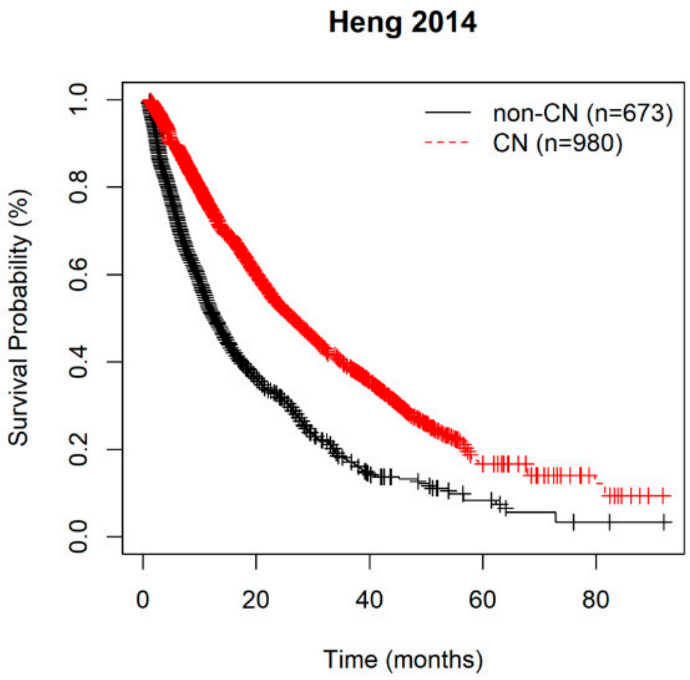
Heng 2014
non-CN (n=673)
CN (n=980)
Survival Probability (%)
0.0
0.2
0.4
0.6
0.8
1.0
0
20
40
60
80
Time (months)

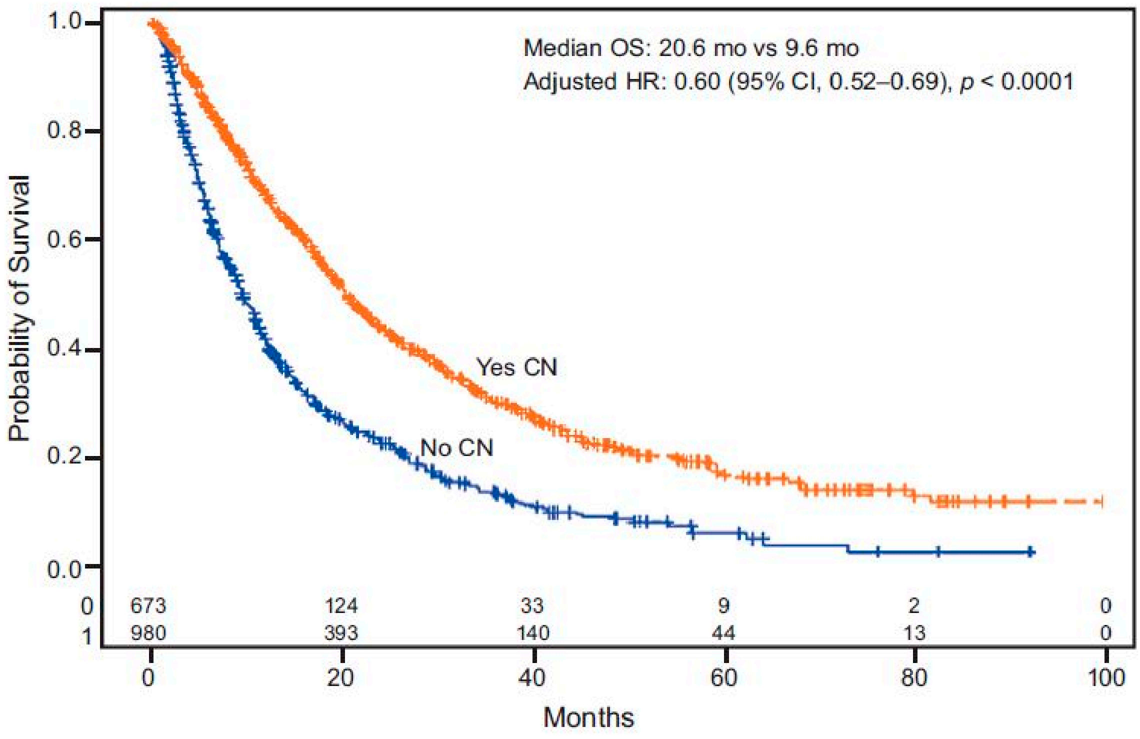
Median OS: 20.6 mo vs 9.6 mo
Adjusted HR: 0.60 (95% CI, 0.52–0.69), p < 0.0001
Yes CN
No CN
Probability of Survival
0.0
0.2
0.4
0.6
0.8
1.0
0 673 124 33 9 2 0
1 980 393 140 44 13 0
0
20
40
60
80
100
Months

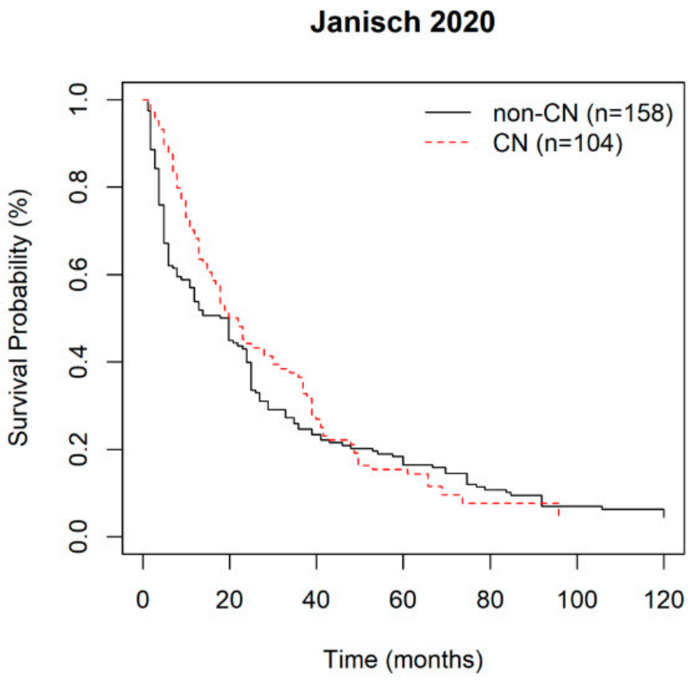
Janisch 2020
non-CN (n=158)
CN (n=104)
Survival Probability (%)
0.0
0.2
0.4
0.6
0.8
1.0
0
20
40
60
80
100
120
Time (months)

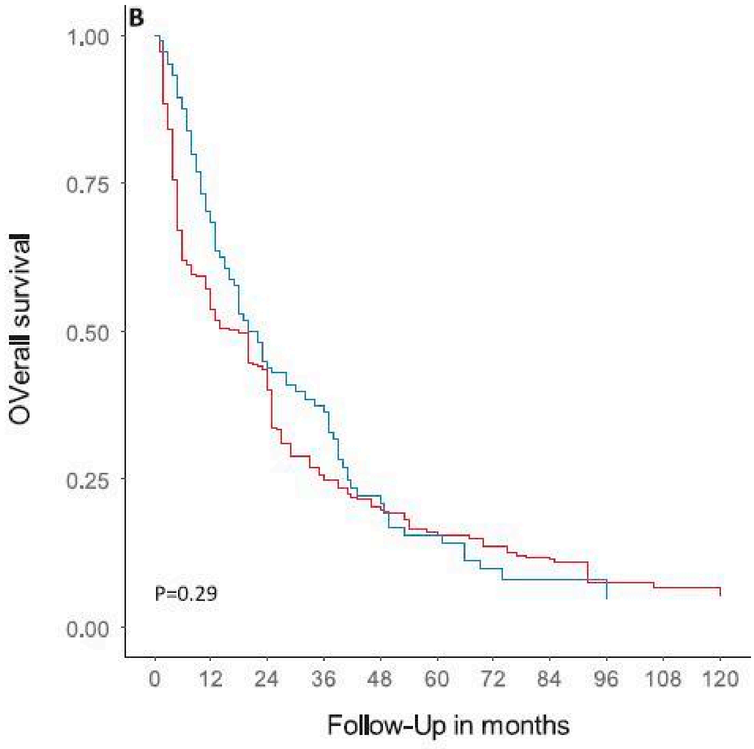
B
OVerall survival
1.00
0.75
0.50
0.25
0.00
P=0.29
0
12
24
36
48
60
72
84
96
108
120
Follow-Up in months

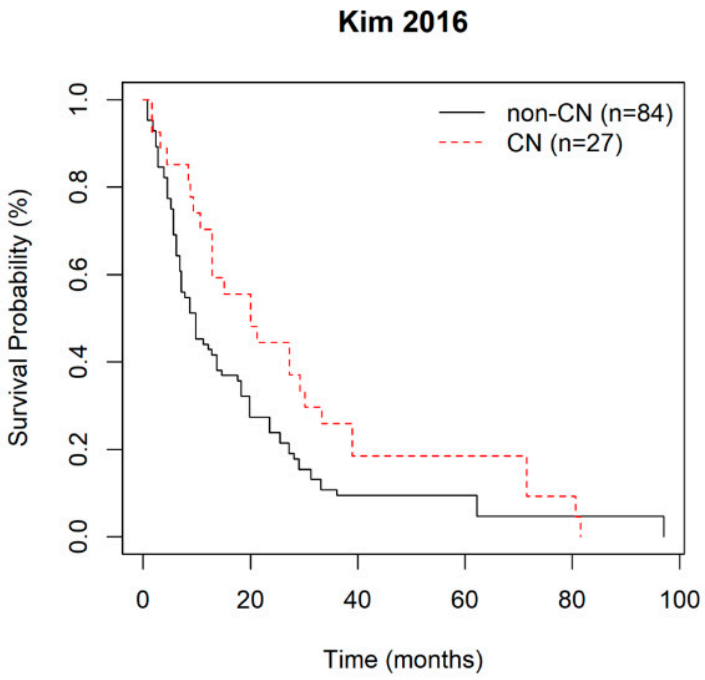
Kim 2016
non-CN (n=84)
CN (n=27)
Survival Probability (%)
Time (months)
0.0
0.2
0.4
0.6
0.8
1.0
0
20
40
60
80
100

D

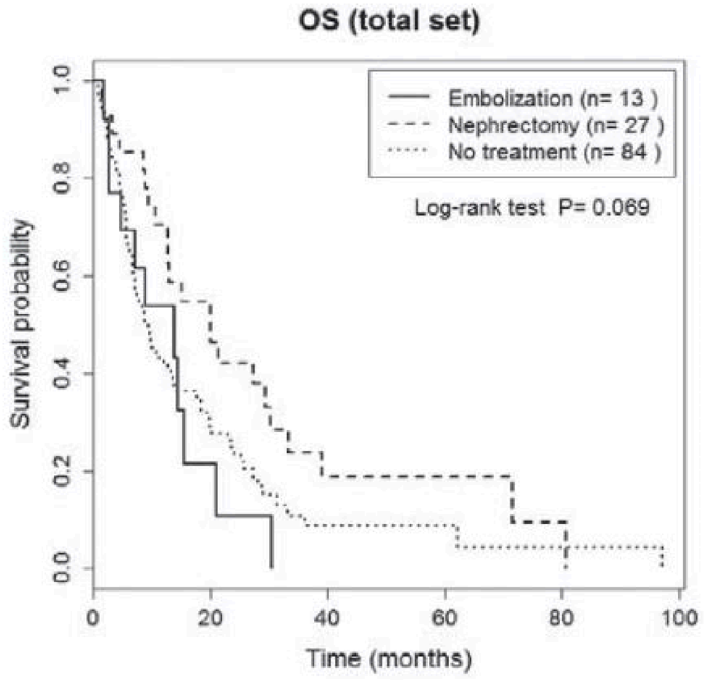
OS (total set)
Embolization (n= 13)
Nephrectomy (n= 27)
No treatment (n= 84)
Log-rank test P= 0.069
Survival probability
Time (months)
0.0
0.2
0.4
0.6
0.8
1.0
0
20
40
60
80
100

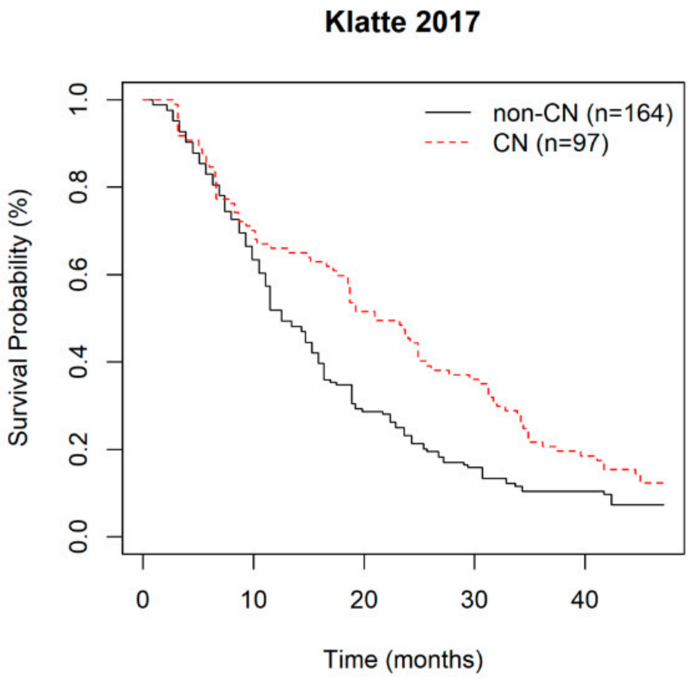
Klatte 2017
non-CN (n=164)
CN (n=97)
Survival Probability (%)
1.0
0.8
0.6
0.4
0.2
0.0
0
10
20
30
40
Time (months)

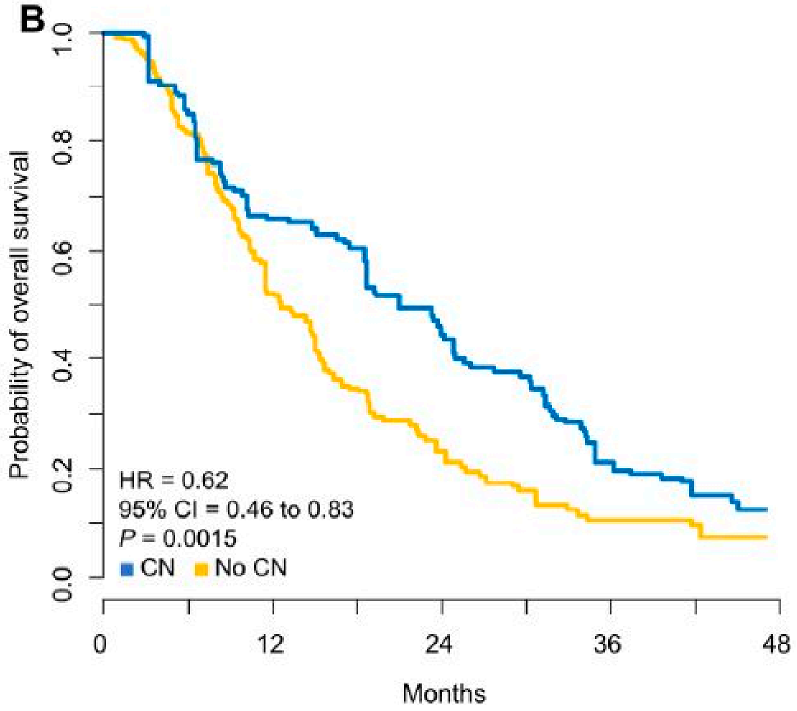
B
Probability of overall survival
1.0
0.8
0.6
0.4
0.2
0.0
HR = 0.62
95% CI = 0.46 to 0.83
P = 0.0015
CN
No CN
0
12
24
36
48
Months

Manley 2017a

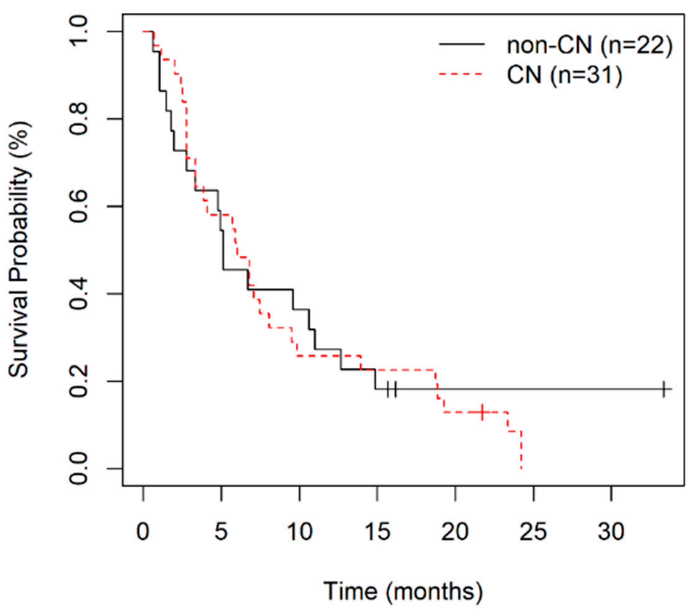

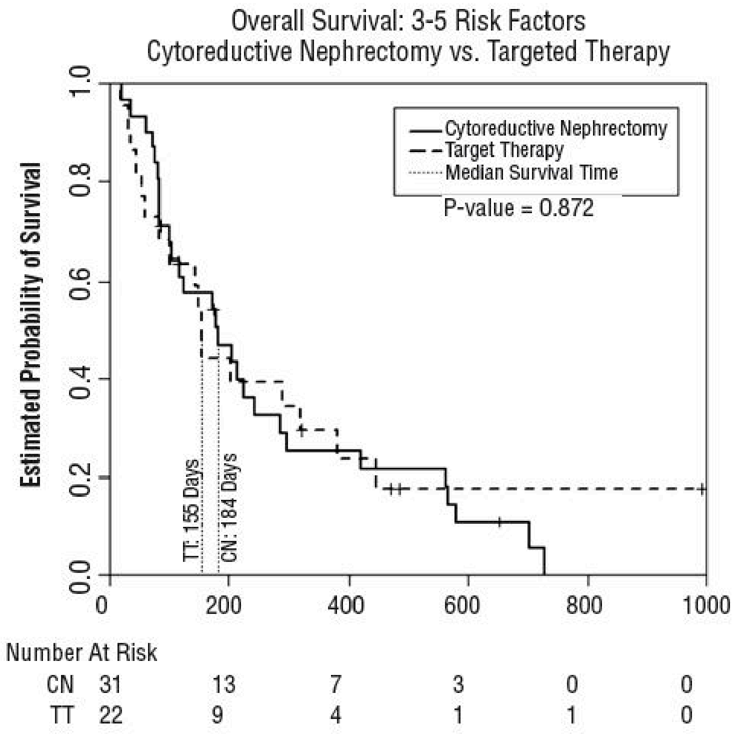

Manley 2017b

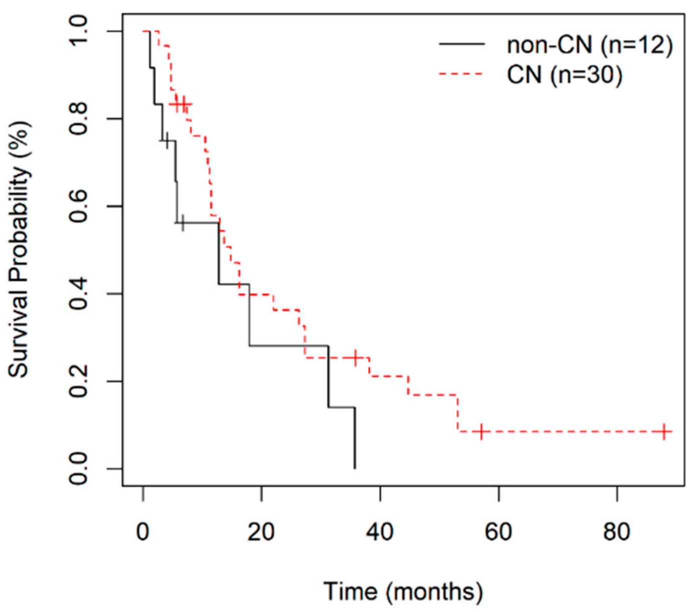

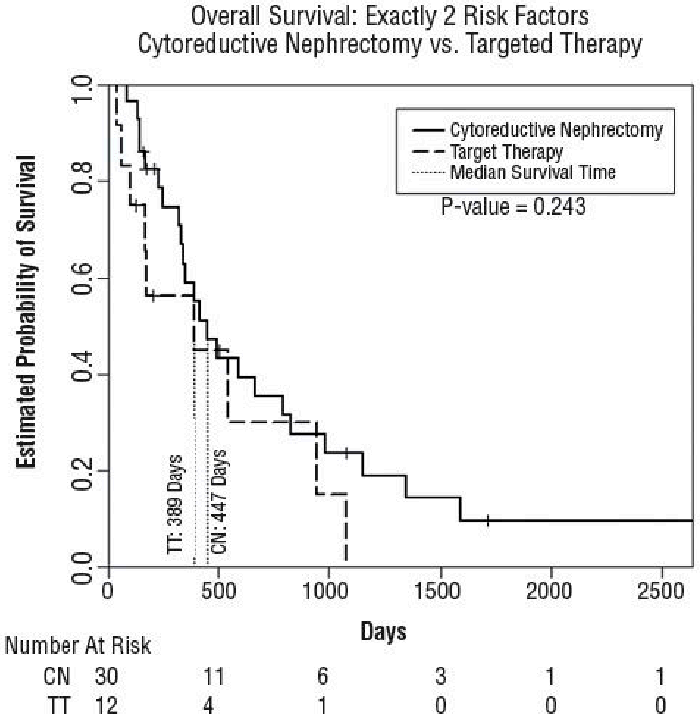

Mejean 2018

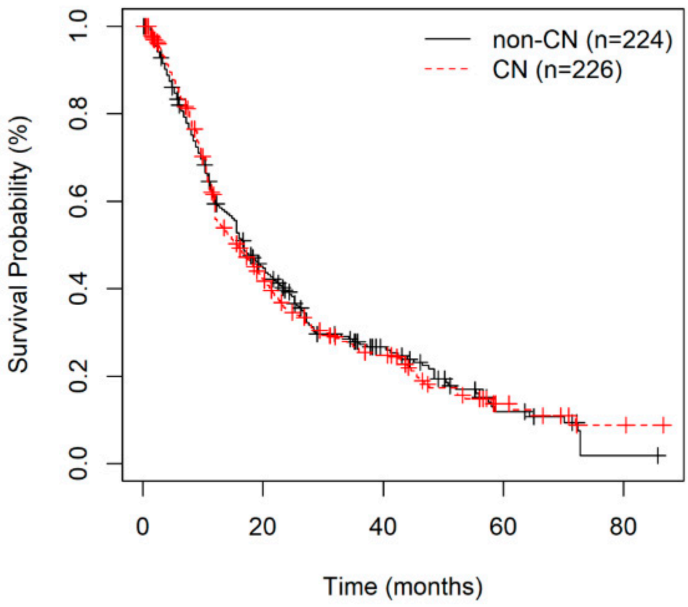

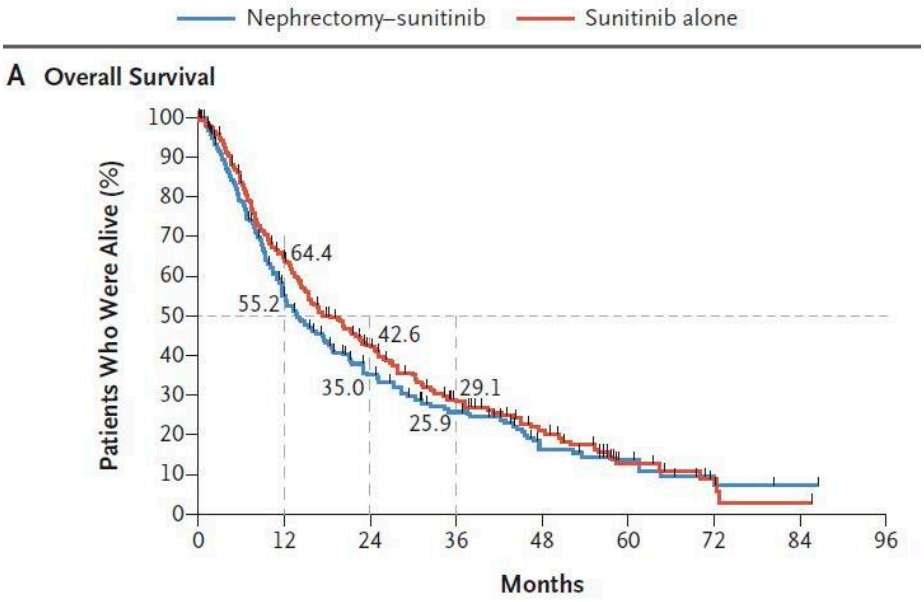

No. at Risk

Nephrectomy–sunitinib	226	110	61	40	19	11	4	1	0
Sunitinib alone	224	128	76	44	26	8	3	1	0

Mutlu 2014

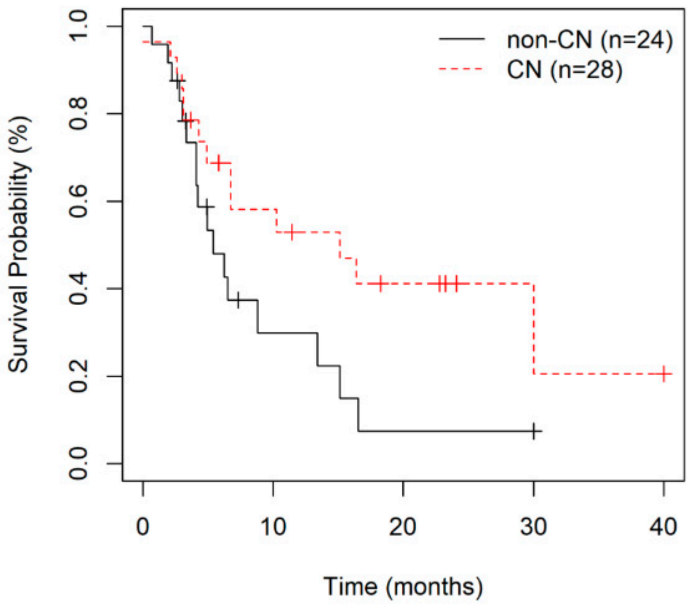
non-CN (n=24)
CN (n=28)
Survival Probability (%)
Time (months)
0.0
0.2
0.4
0.6
0.8
1.0
0
10
20
30
40

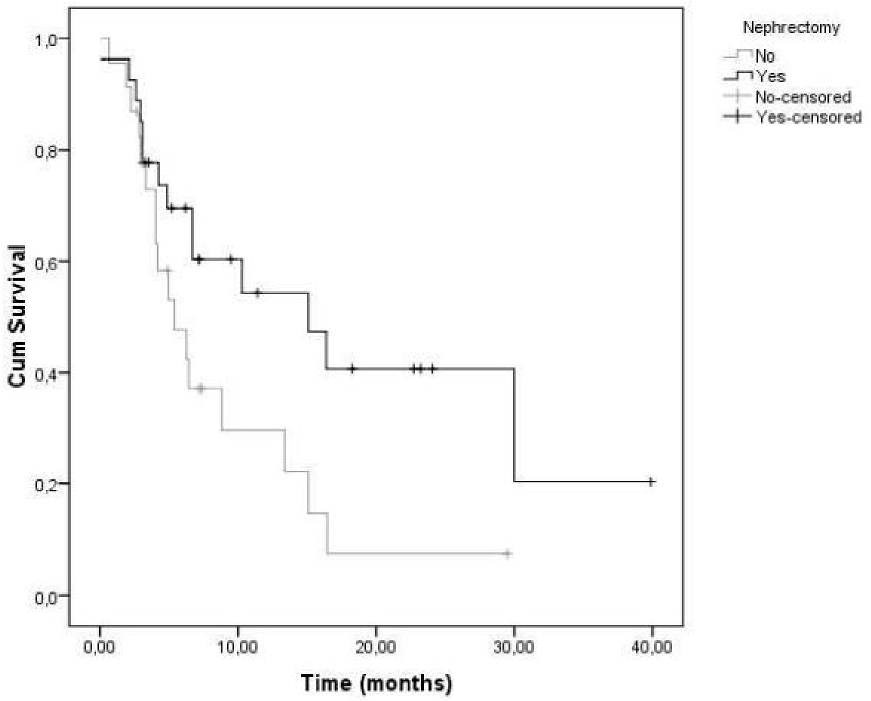
Nephrectomy
No
Yes
No-censored
Yes-censored
Cum Survival
Time (months)
0,0
0,2
0,4
0,6
0,8
1,0
0,00
10,00
20,00
30,00
40,00

Poprach 2018

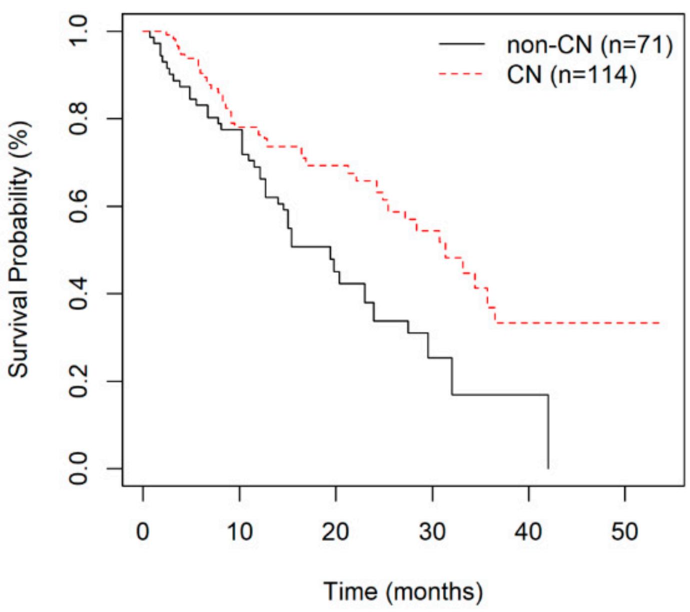

B

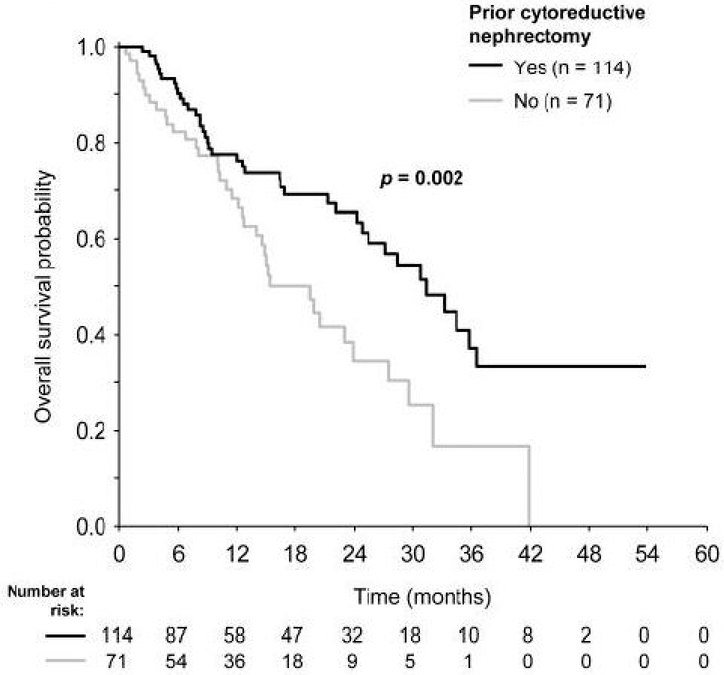

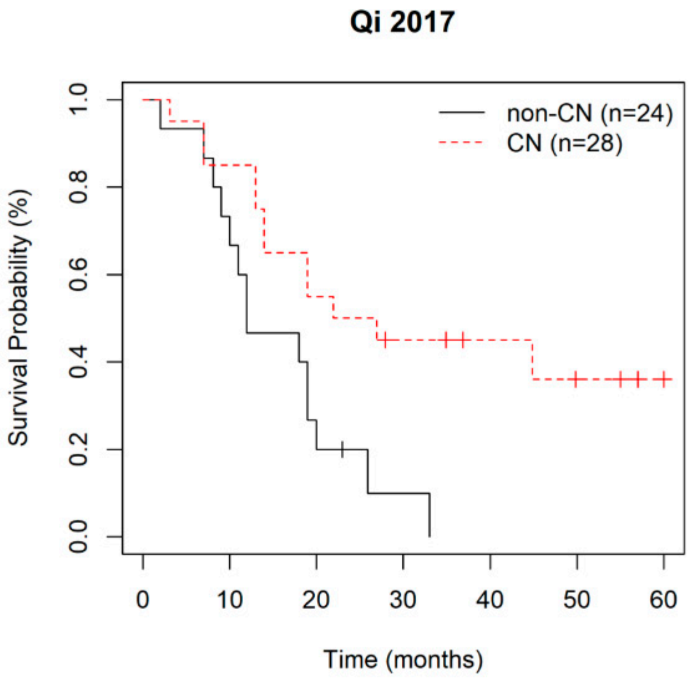

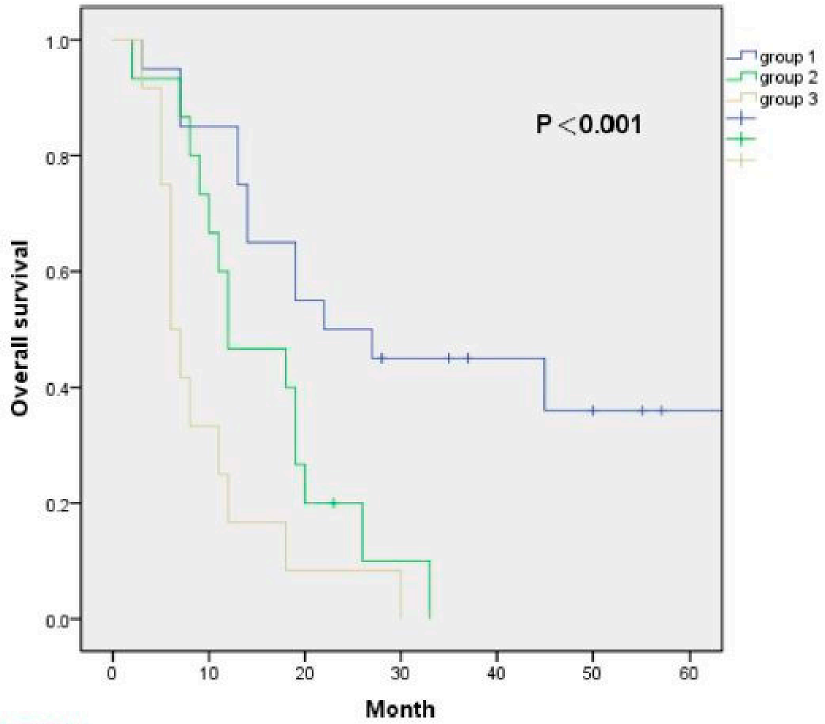

No. at risk							
Group 1	20	17	11	7	5	3	1
Group 2	15	10	3	1	0		

Song 2016

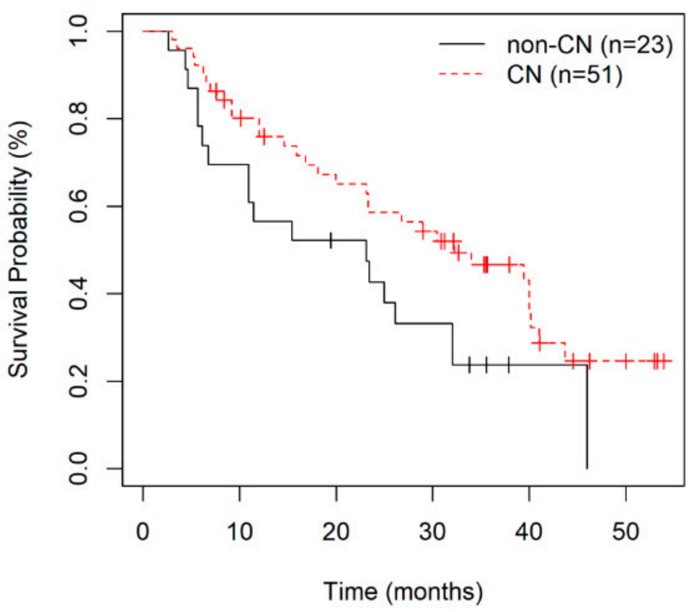

non-CN (n=23)
CN (n=51)
Survival Probability (%)
Time (months)

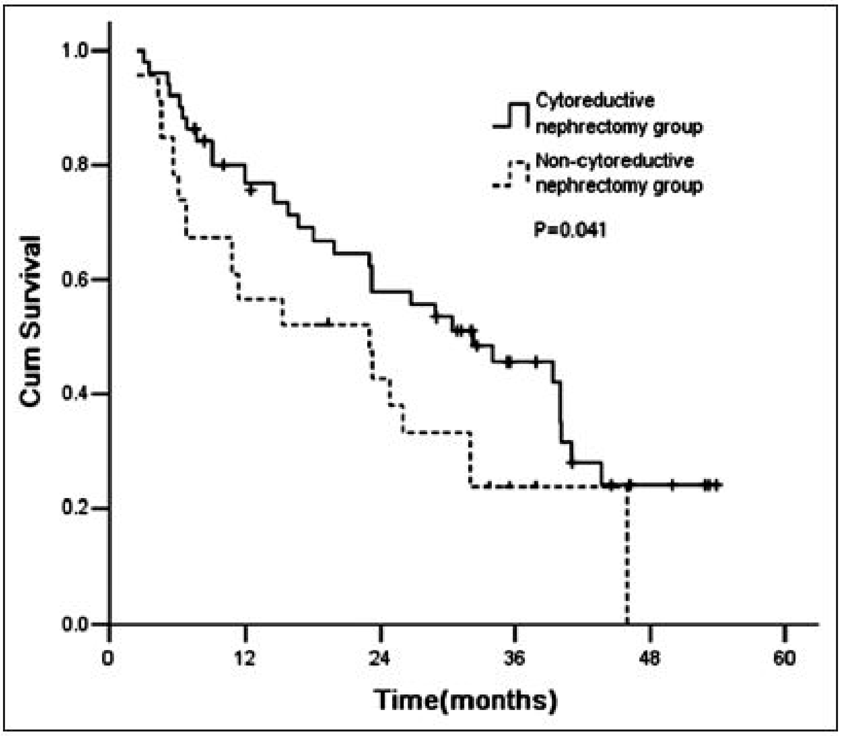

Cytoreductive nephrectomy group
Non-cytoreductive nephrectomy group
P=0.041
Cum Survival
Time(months)

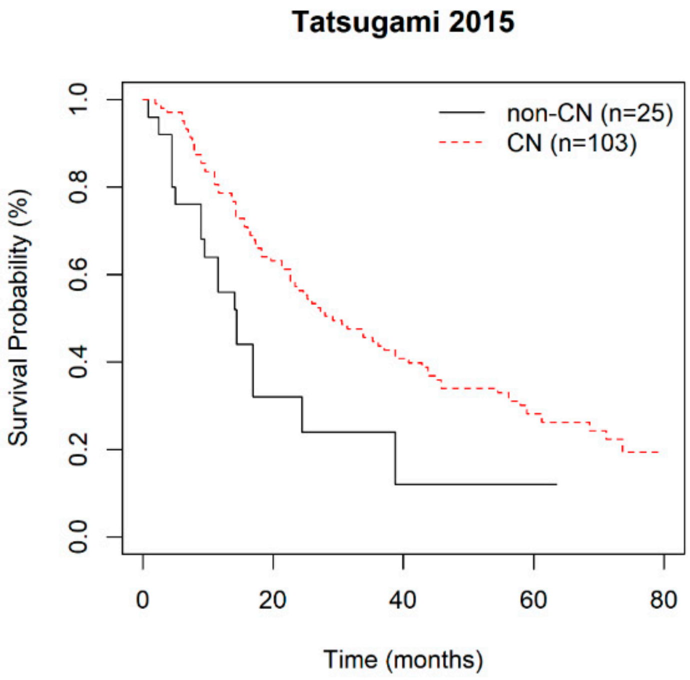

Tatsugami 2015
non-CN (n=25)
CN (n=103)
Survival Probability (%)
0.0
0.2
0.4
0.6
0.8
1.0
0
20
40
60
80
Time (months)

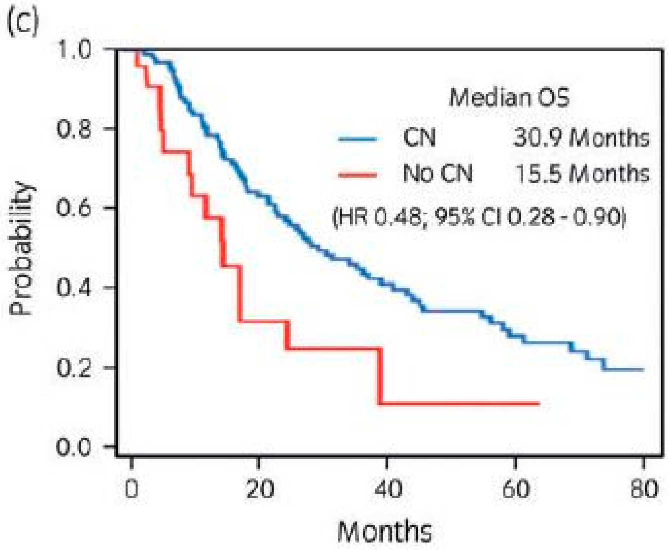

(c)
Median OS
CN 30.9 Months
No CN 15.5 Months
(HR 0.48; 95% CI 0.28 - 0.90)
Probability
0.0
0.2
0.4
0.6
0.8
1.0
0
20
40
60
80
Months

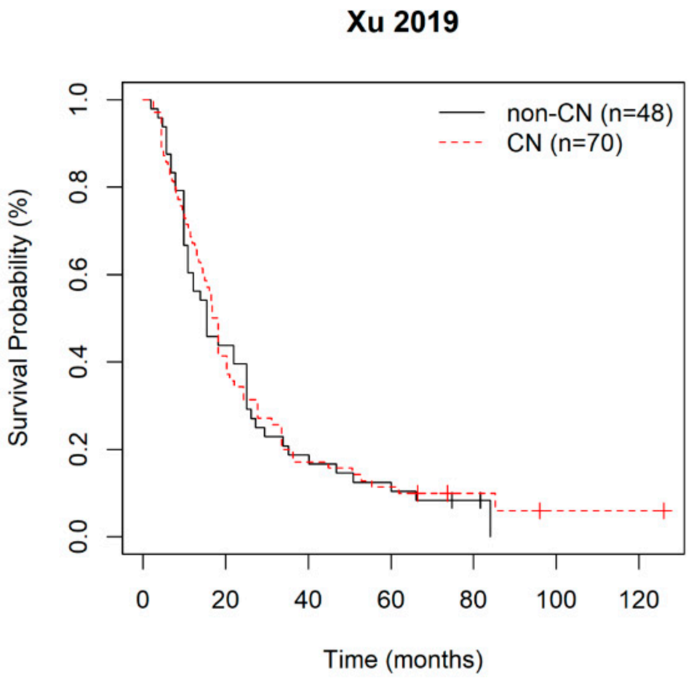
Xu 2019
Survival Probability (%)
non-CN (n=48)
CN (n=70)
0.0
0.2
0.4
0.6
0.8
1.0
0
20
40
60
80
100
120
Time (months)

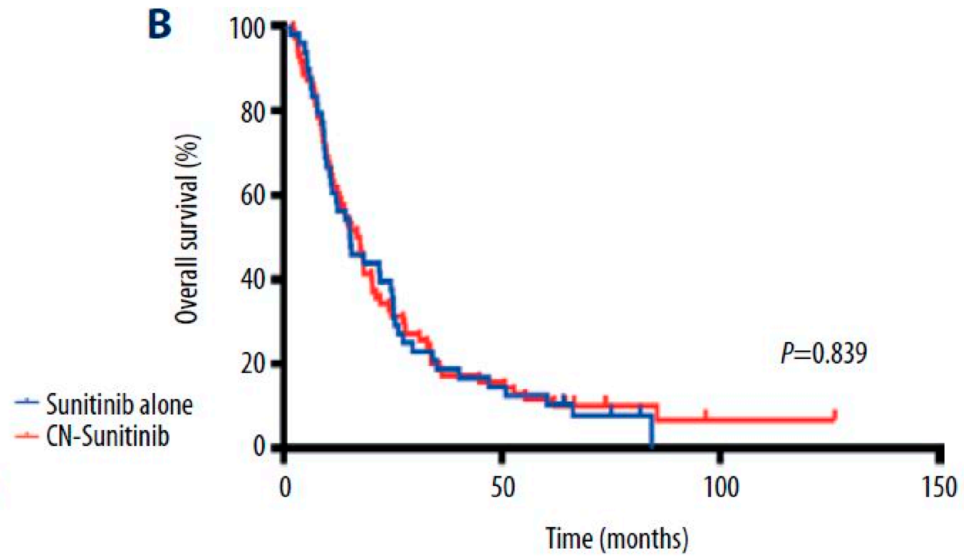
B
Overall survival (%)
100
80
60
40
20
0
P=0.839
Sunitinib alone
CN-Sunitinib
0
50
100
150
Time (months)

You 2015

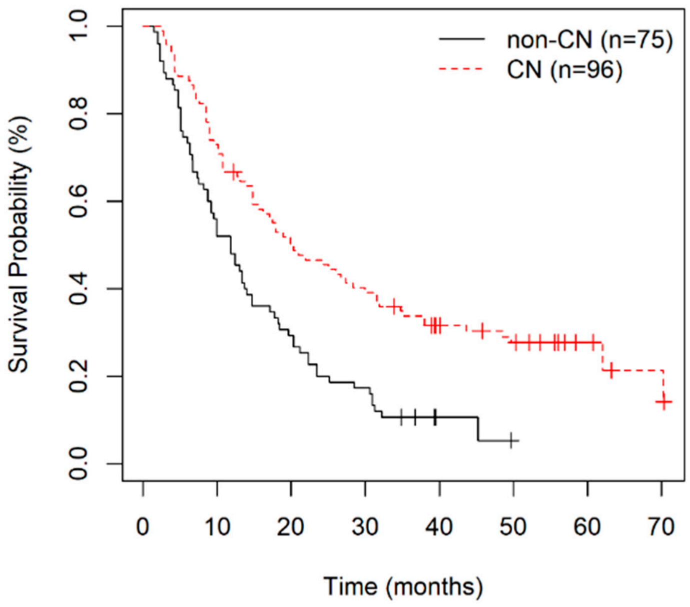

non-CN (n=75)
CN (n=96)
Survival Probability (%)
0.0
0.2
0.4
0.6
0.8
1.0
0
10
20
30
40
50
60
70
Time (months)

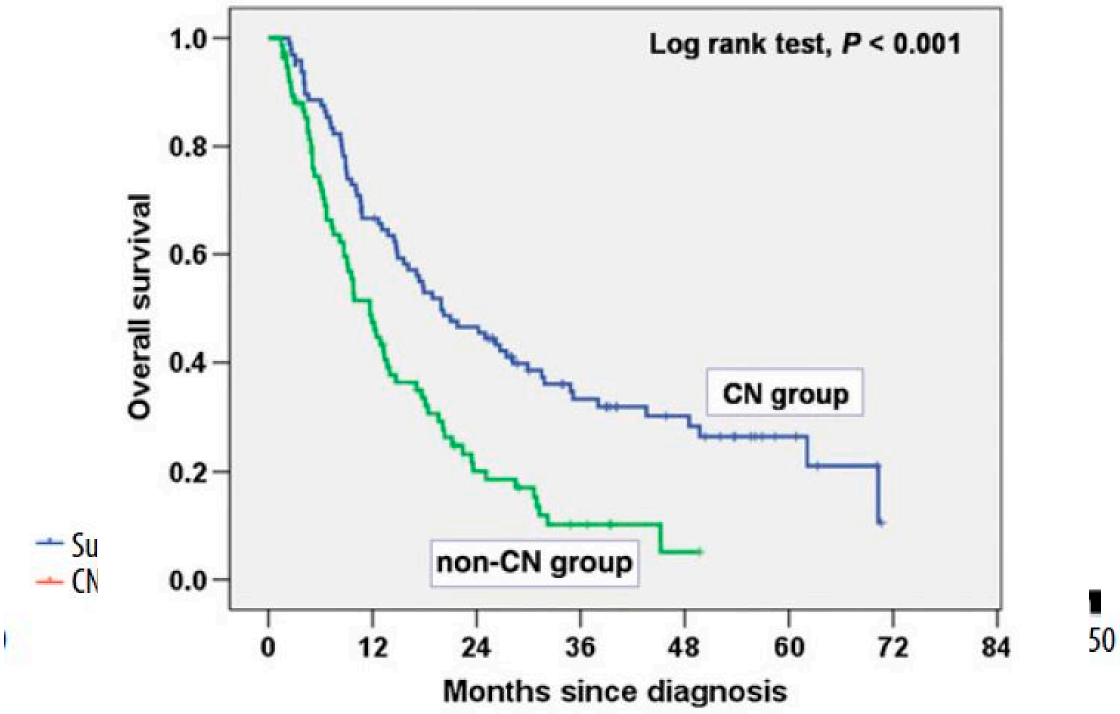

Log rank test, *P* < 0.001
CN group
non-CN group
Overall survival
1.0
0.8
0.6
0.4
0.2
0.0
0
12
24
36
48
60
72
84
Months since diagnosis
Su
CN
50

Progression-Free Survival

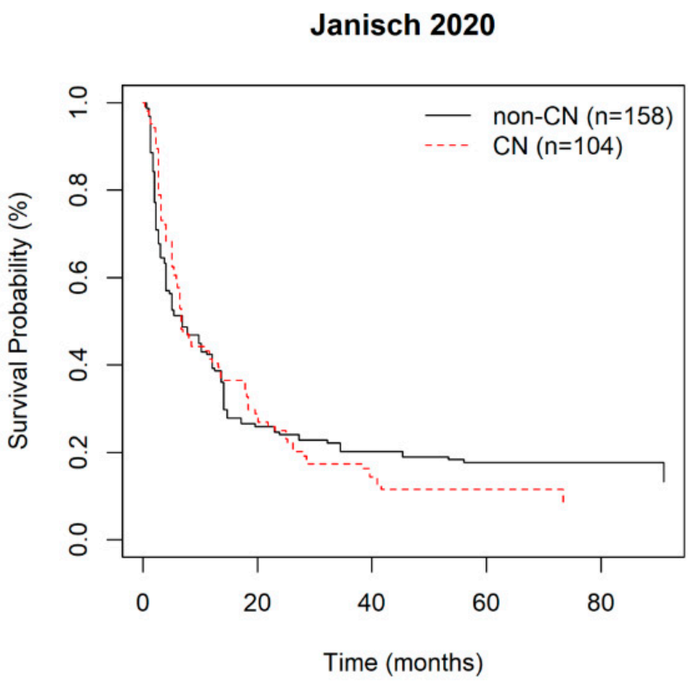

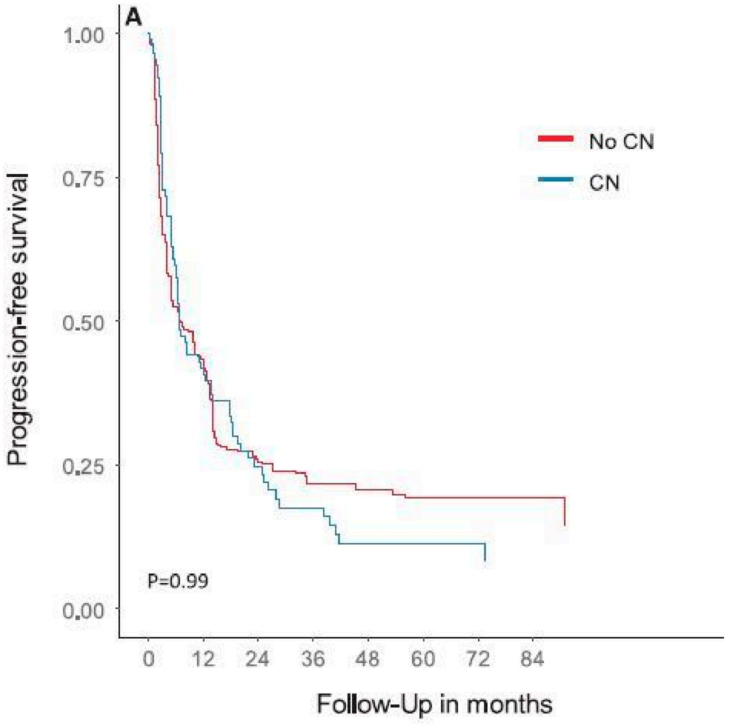

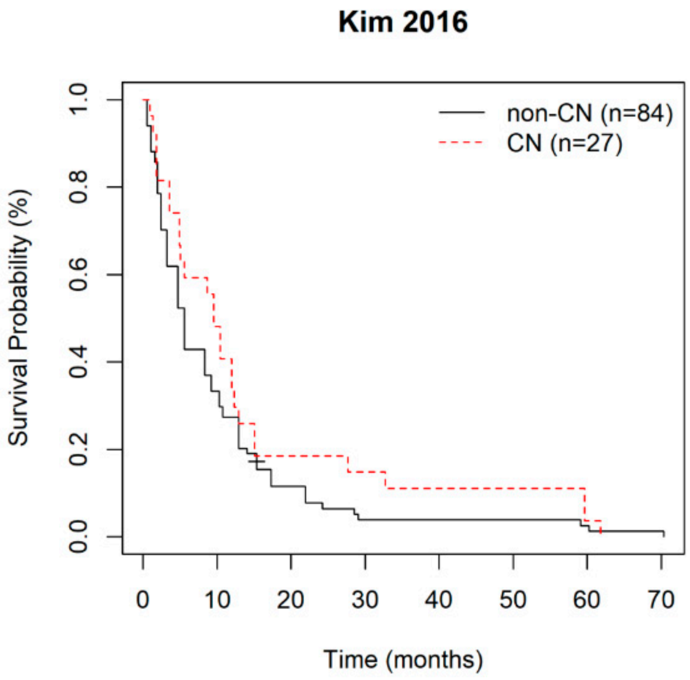
Kim 2016
non-CN (n=84)
CN (n=27)
Survival Probability (%)
1.0
0.8
0.6
0.4
0.2
0.0
0
10
20
30
40
50
60
70
Time (months)

C

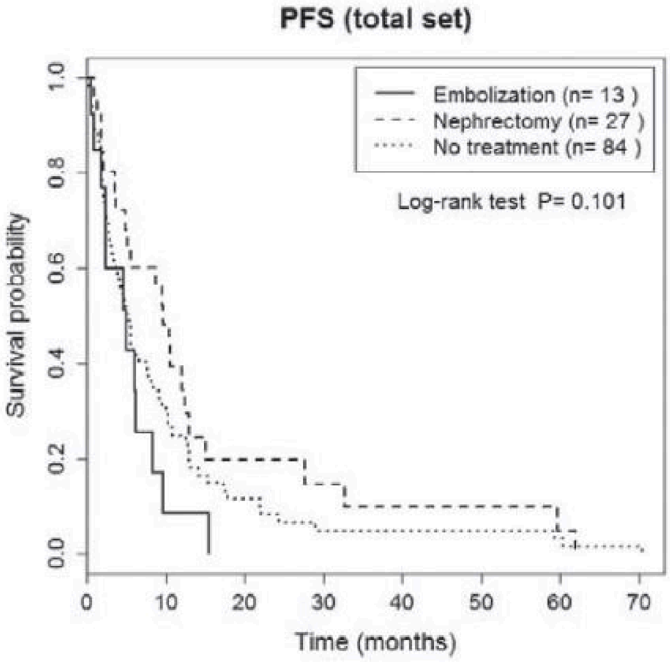
PFS (total set)
Embolization (n= 13)
Nephrectomy (n= 27)
No treatment (n= 84)
Log-rank test P= 0.101
Survival probability
1.0
0.8
0.6
0.4
0.2
0.0
0
10
20
30
40
50
60
70
Time (months)

Mejean 2018

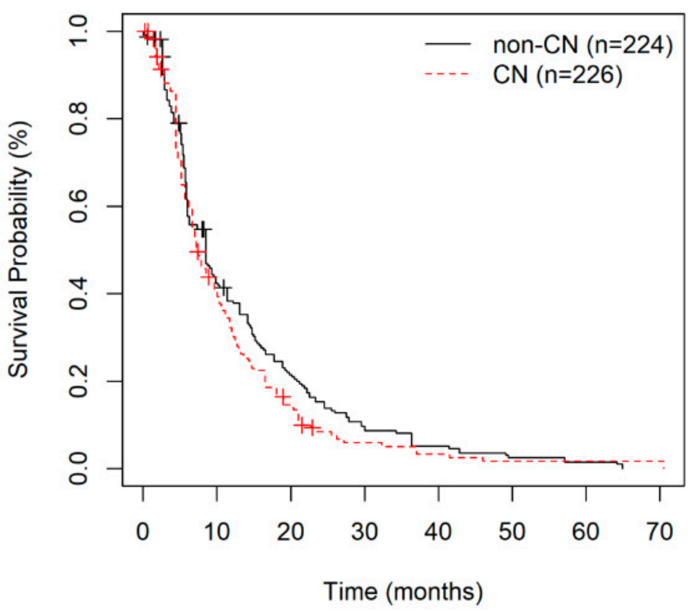

B Progression-free Survival

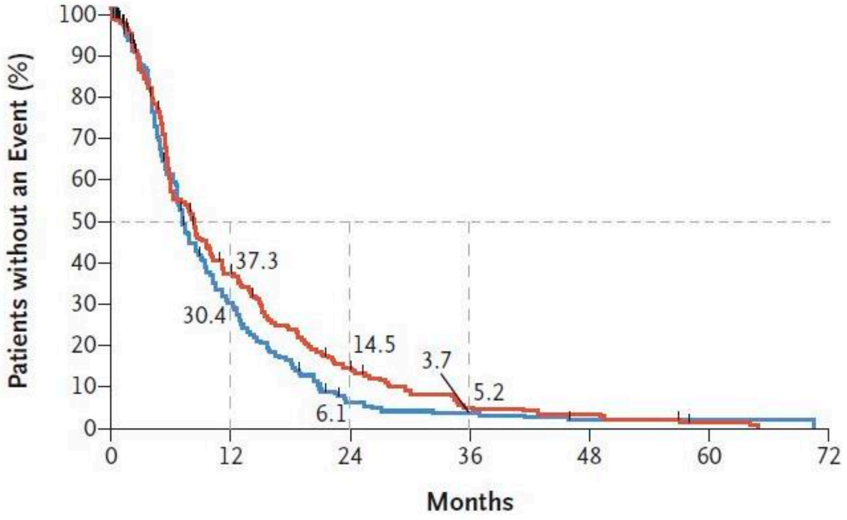

No. at Risk

Nephrectomy–sunitinib	226	59	10	6	2	1	0
Sunitinib alone	224	74	28	9	6	2	0

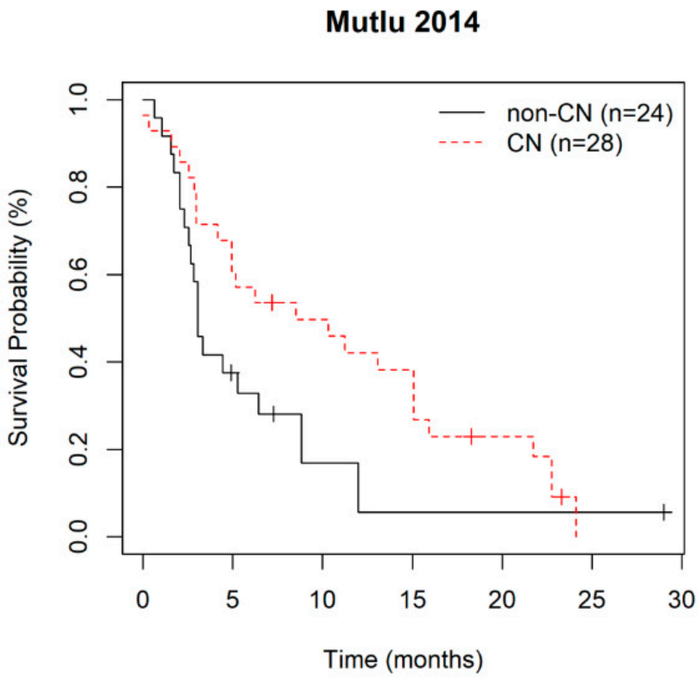

Mutlu 2014
Survival Probability (%)
1.0
0.8
0.6
0.4
0.2
0.0
non-CN (n=24)
CN (n=28)
0
5
10
15
20
25
30
Time (months)

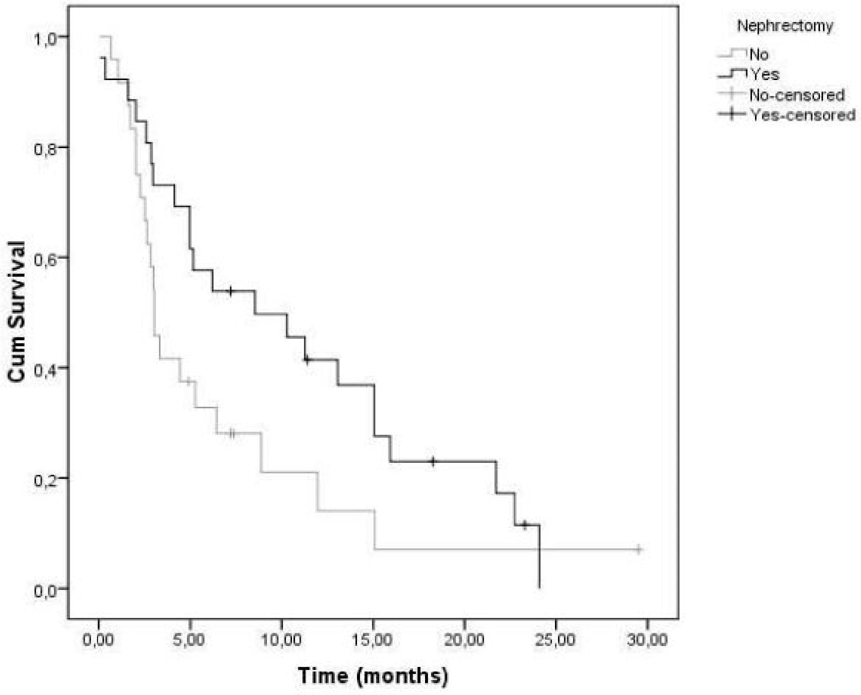

Nephrectomy
No
Yes
No-censored
Yes-censored
Cum Survival
1,0
0,8
0,6
0,4
0,2
0,0
0,00
5,00
10,00
15,00
20,00
25,00
30,00
Time (months)

Poprach 2018

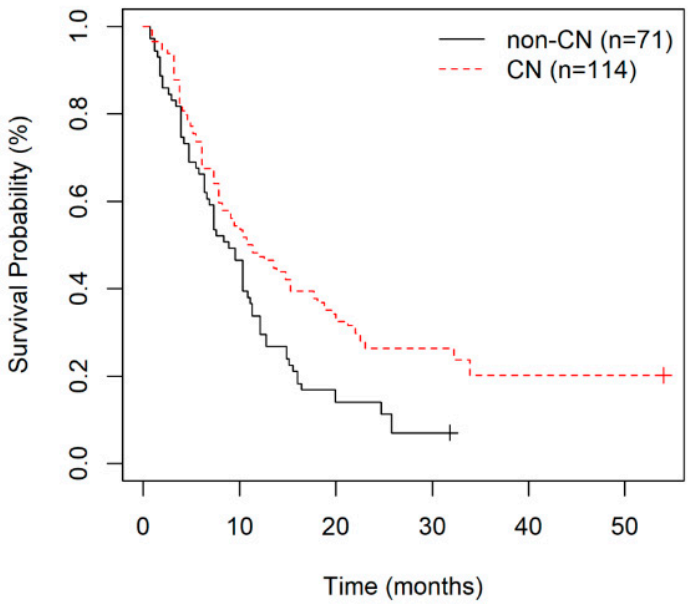

A

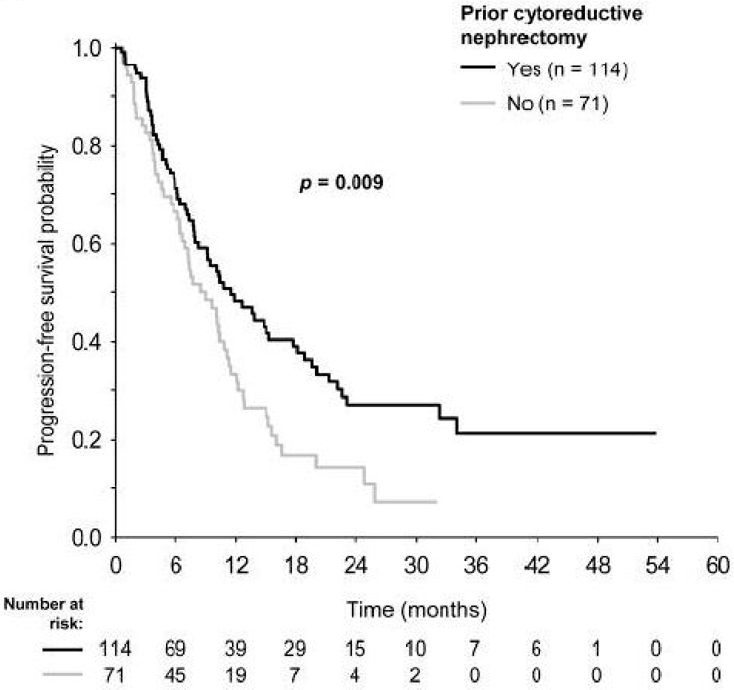

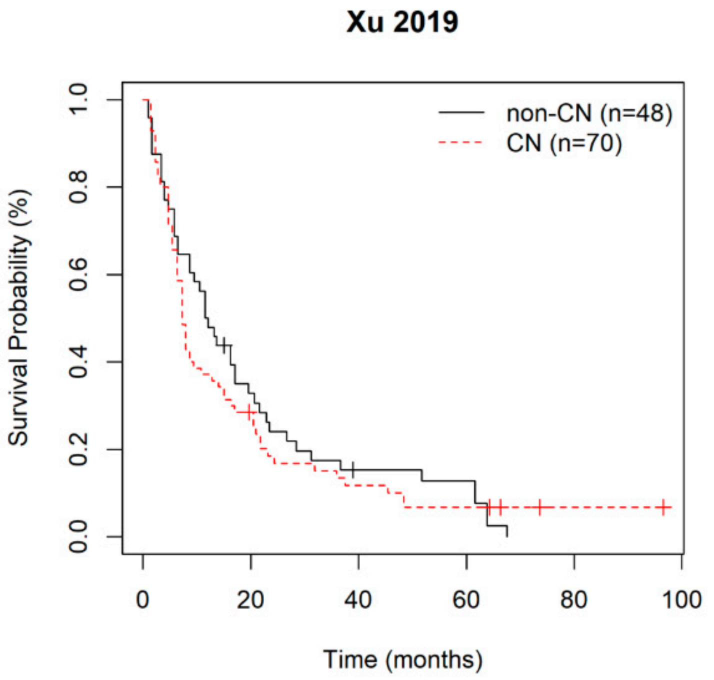
Xu 2019
non-CN (n=48)
CN (n=70)
Survival Probability (%)
0.0
0.2
0.4
0.6
0.8
1.0
0
20
40
60
80
100
Time (months)

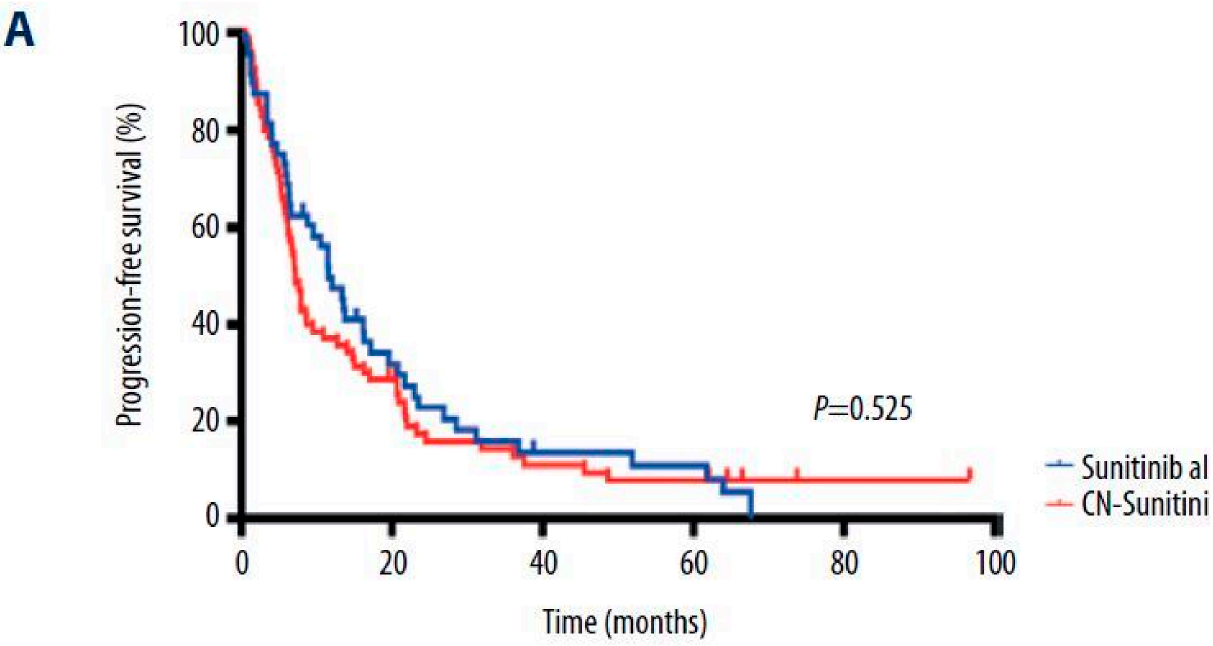
A
Progression-free survival (%)
0
20
40
60
80
100
P=0.525
Sunitinib al
CN-Sunitini
Time (months)

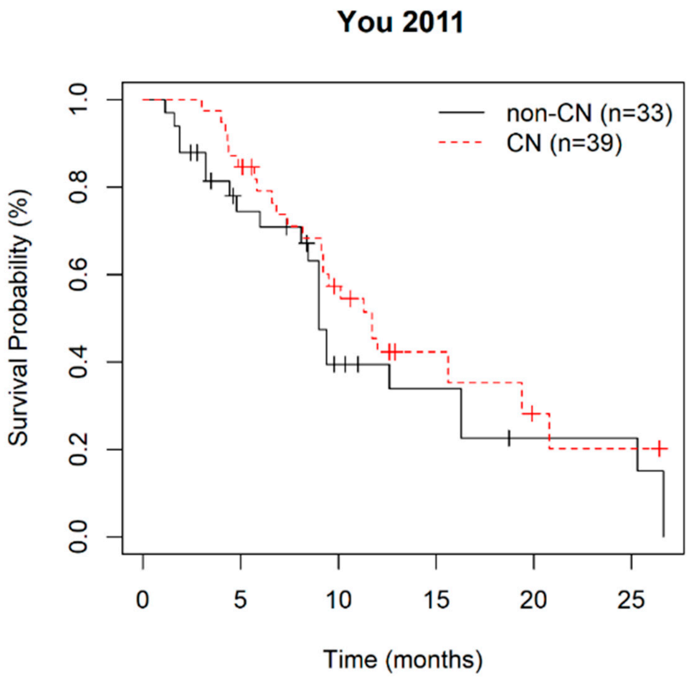
You 2011
non-CN (n=33)
CN (n=39)
Survival Probability (%)
Time (months)
0.0
0.2
0.4
0.6
0.8
1.0
0
5
10
15
20
25

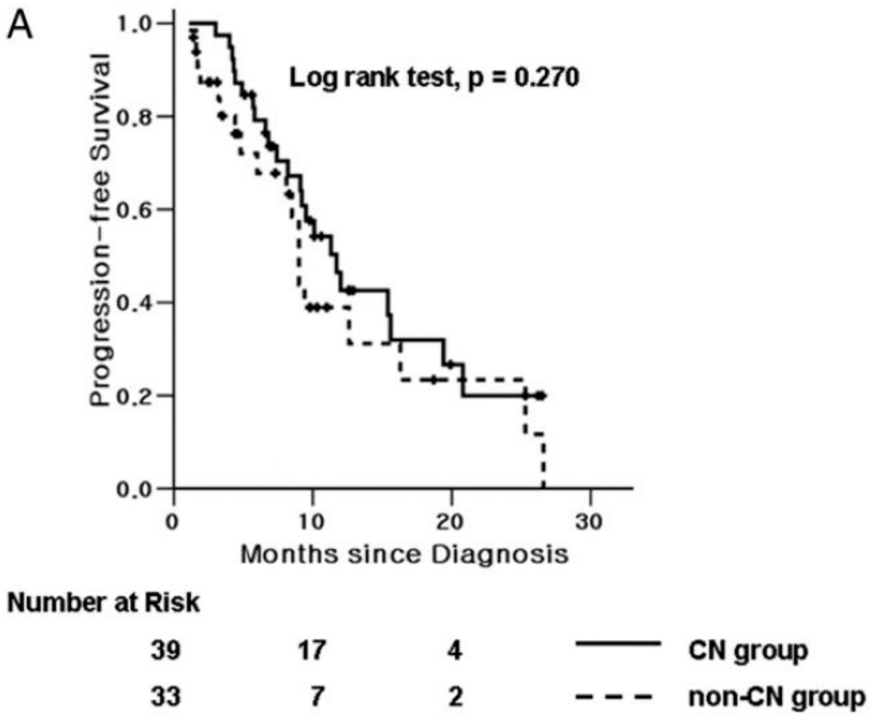
A
Log rank test, p = 0.270
Progression-free Survival
Months since Diagnosis
0.0
0.2
0.4
0.6
0.8
1.0
0
10
20
30
Number at Risk
39 17 4
33 7 2
CN group
non-CN group

Cancer-Specific Survival

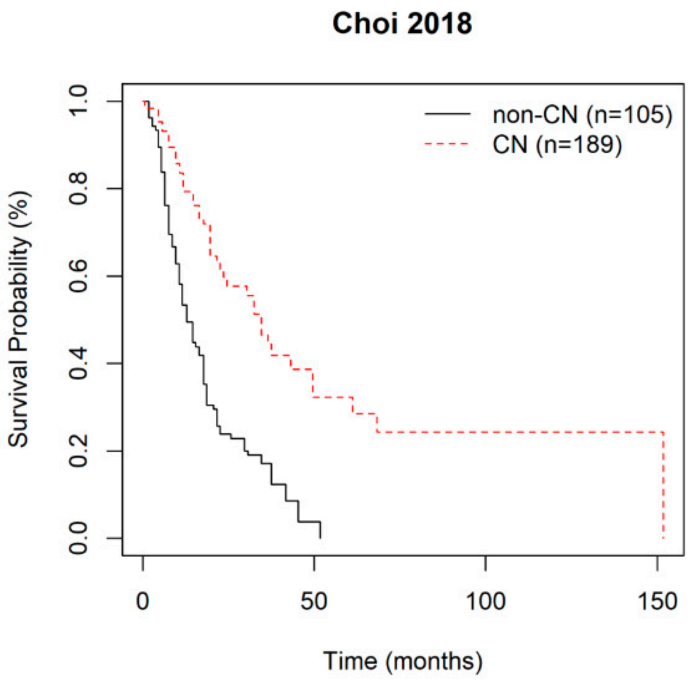

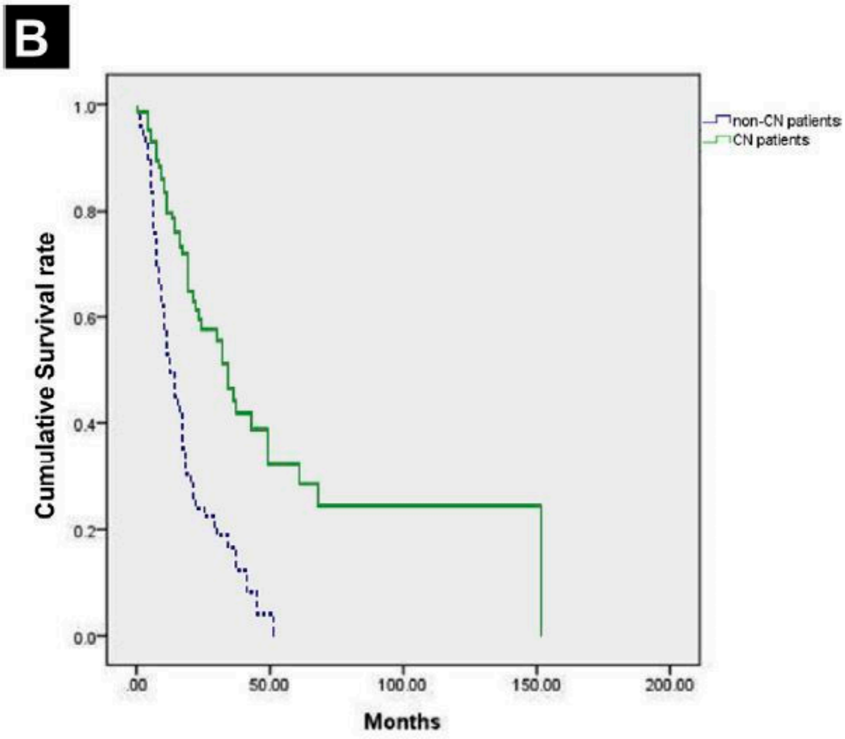

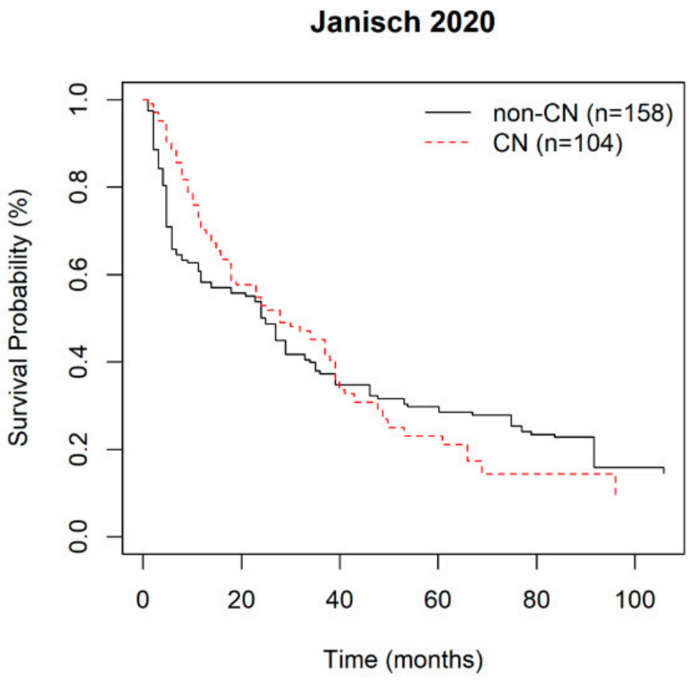
Janisch 2020
non-CN (n=158)
CN (n=104)
Survival Probability (%)
0.0
0.2
0.4
0.6
0.8
1.0
0
20
40
60
80
100
Time (months)

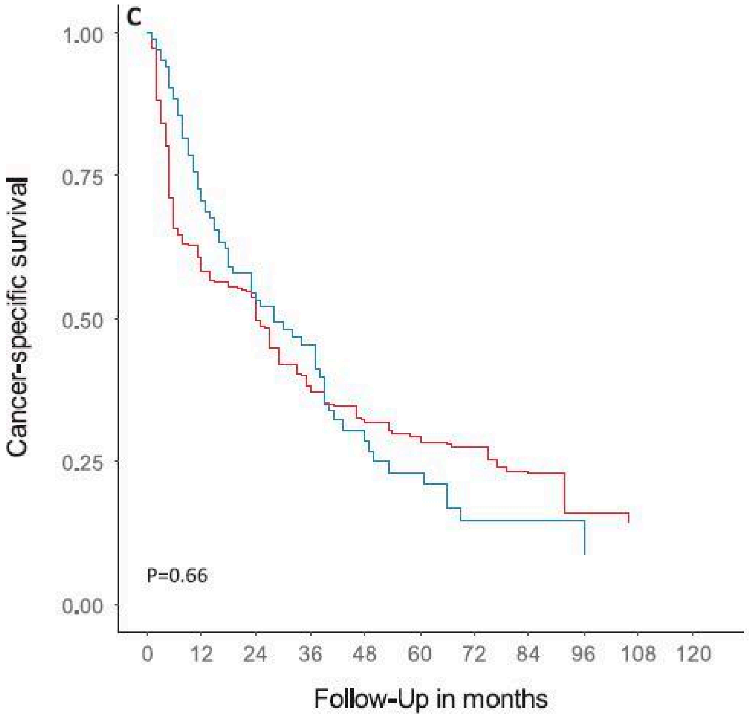
C
1.00
0.75
0.50
0.25
0.00
Cancer-specific survival
P=0.66
0
12
24
36
48
60
72
84
96
108
120
Follow-Up in months

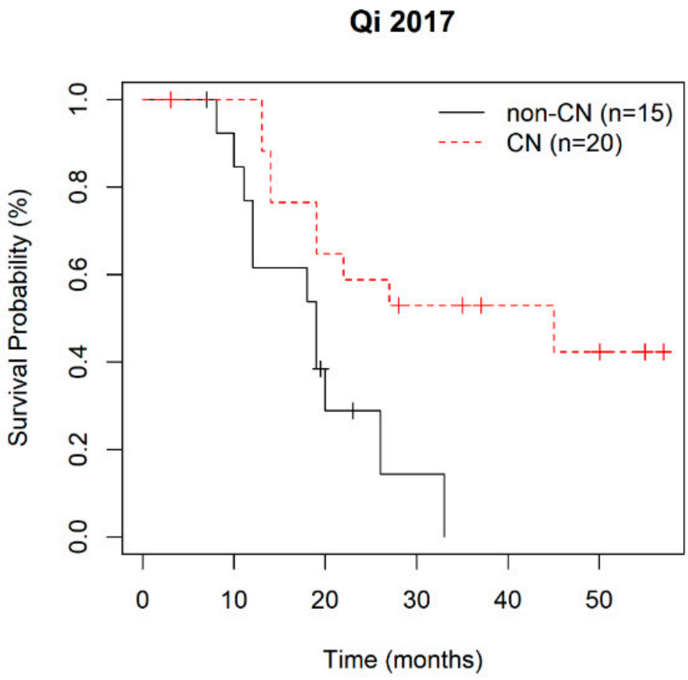
Qi 2017
non-CN (n=15)
CN (n=20)
Survival Probability (%)
Time (months)
0.0
0.2
0.4
0.6
0.8
1.0
0
10
20
30
40
50

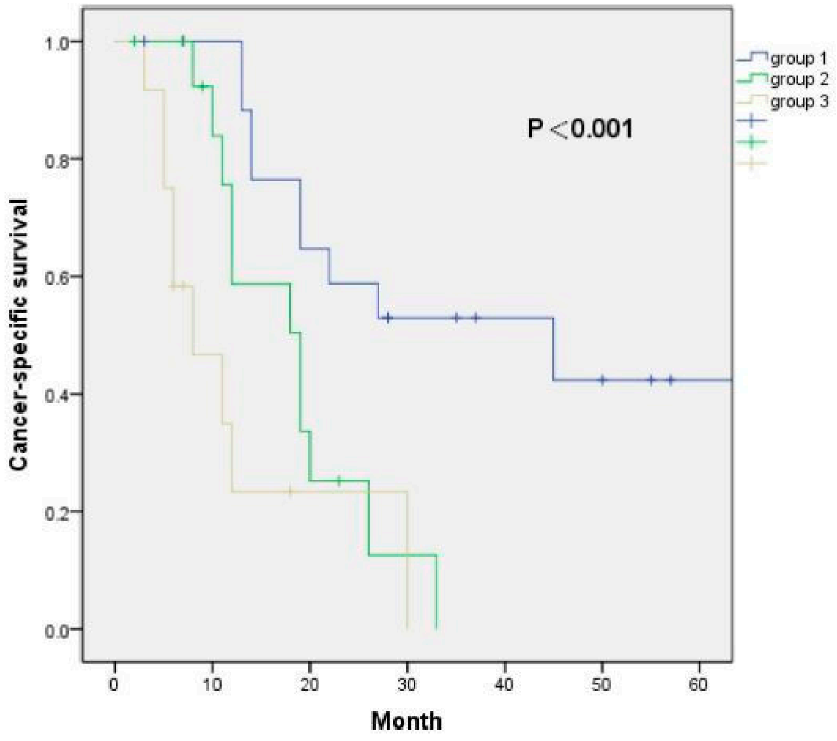
group 1
group 2
group 3
P＜0.001
Cancer-specific survival
Month
0.0
0.2
0.4
0.6
0.8
1.0
0
10
20
30
40
50
60
