# Supplementary material for: Long-Term Survival Outcomes of Cytoreductive Nephrectomy Combined with Targeted Therapy for Metastatic Renal Cell Carcinoma: A Systematic Review and Individual Patient Data Meta-Analysis
Source: Cancers (Basel). 2021 Feb 9;13(4):695. doi: 10.3390/cancers13040695 (PMC7915816; doi:10.3390/cancers13040695)

Supplemental File 4. Visual evaluation of the proportionality-of-hazards assumption.

Evaluation of proportional hazards assumption using scaled Schoenfeld residuals versus time (Overall Survival)

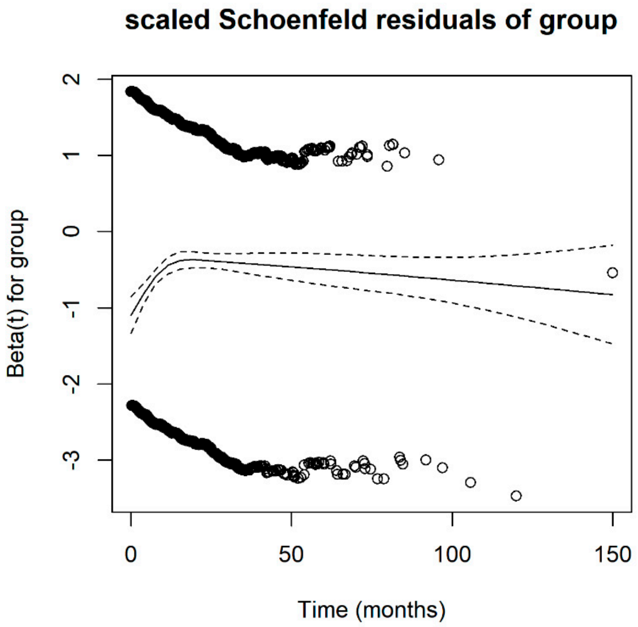

Evaluation of proportional hazards assumption using scaled Schoenfeld residuals versus time (Progression-Free Survival)

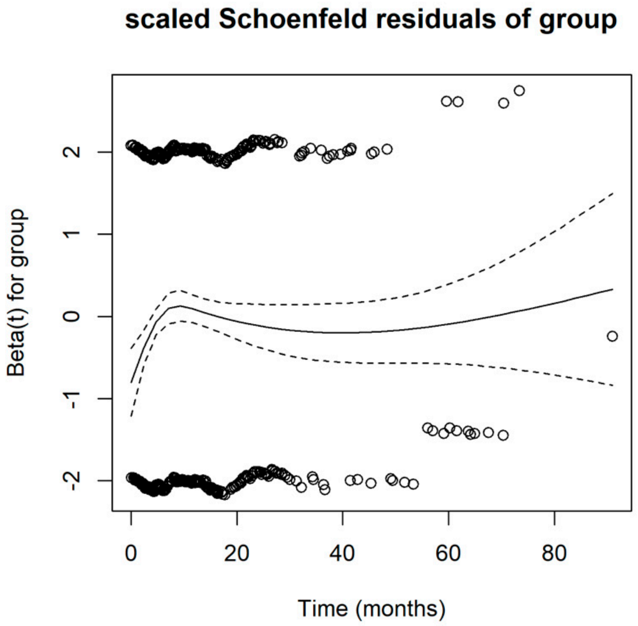

Evaluation of proportional hazards assumption using scaled Schoenfeld residuals versus time (Cancer-Specific Survival)

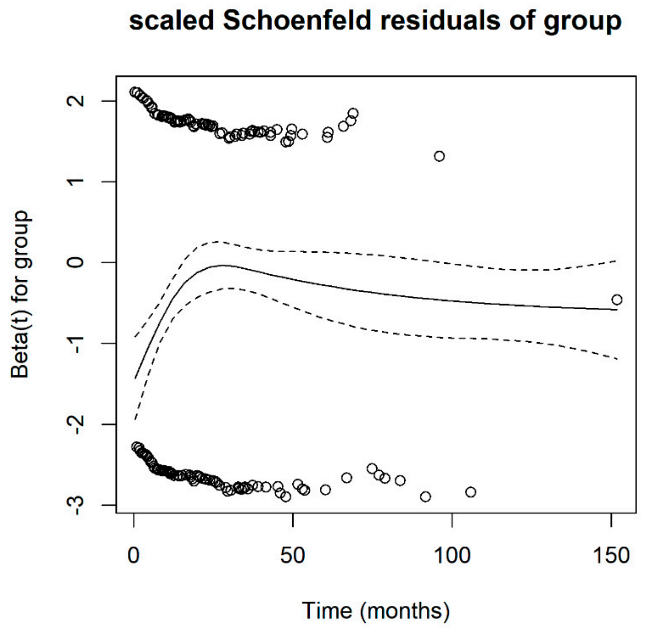

Assessment of proportional hazards assumption using log-log plot of survivor functions (Overall Survival)

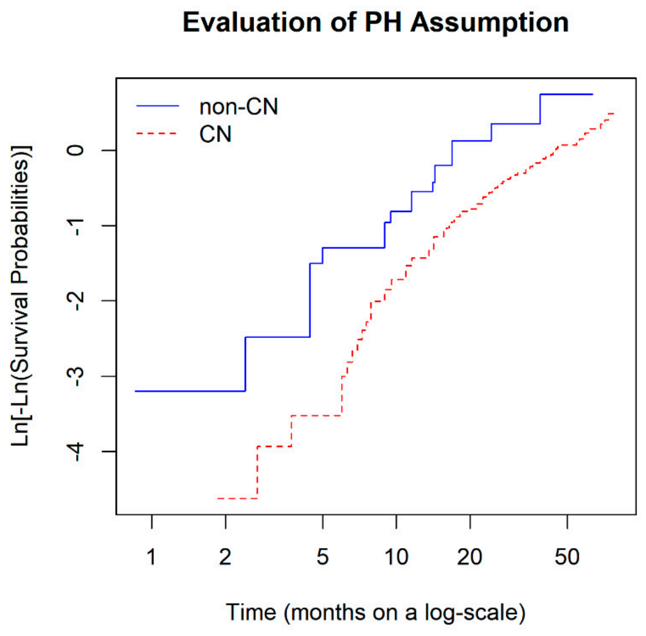

Assessment of proportional hazards assumption using log-log plot of survivor functions (Progression-Free Survival)

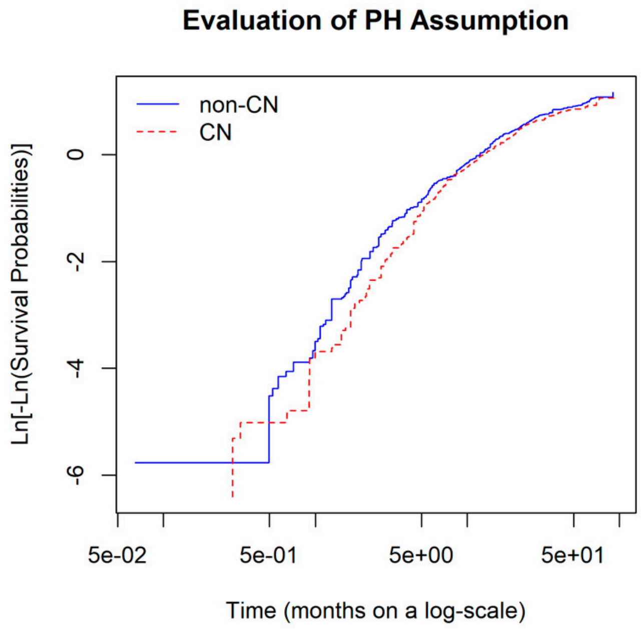

Assessment of proportional hazards assumption using log-log plot of survivor functions (Cancer-Specific Survival)

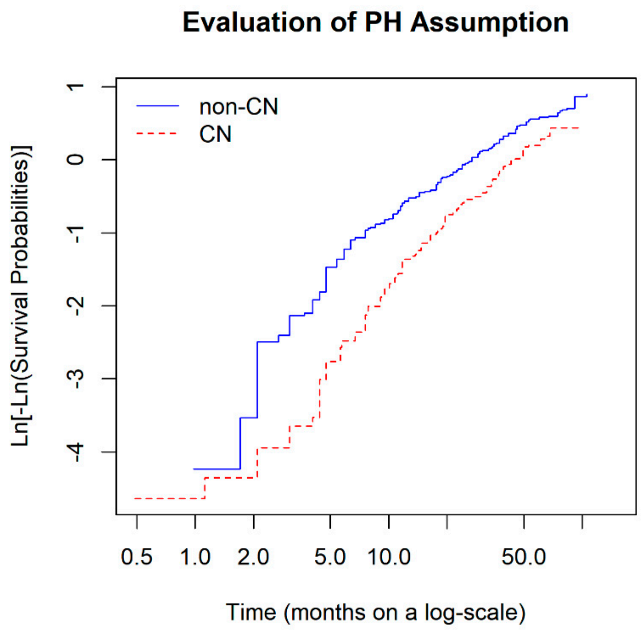

Assessment of proportional hazards assumption using fitted versus predicted survival functions (Overall Survival)

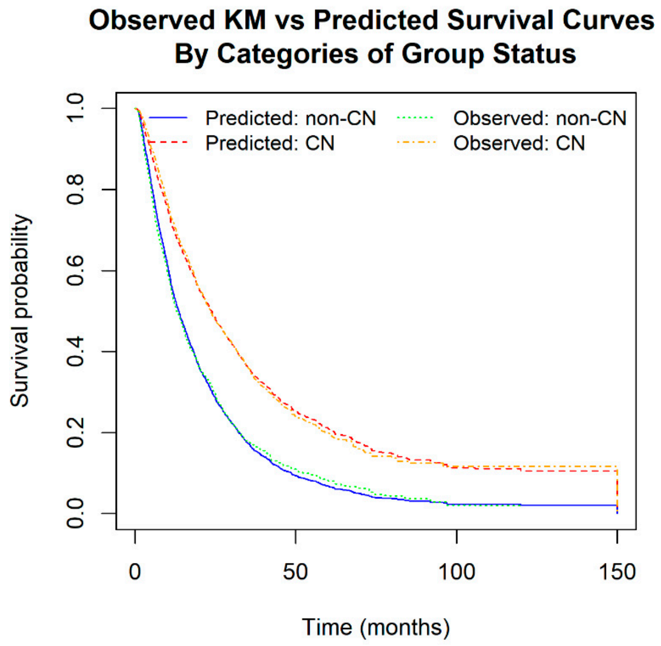

Assessment of proportional hazards assumption using fitted versus predicted survival functions (Progression-Free Survival)

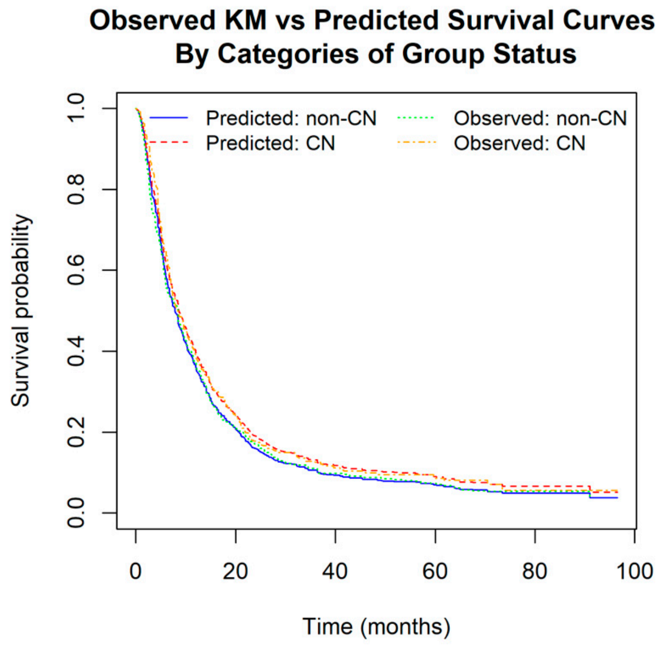

Assessment of proportional hazards assumption using fitted versus predicted survival functions (Cancer-Specific Survival)

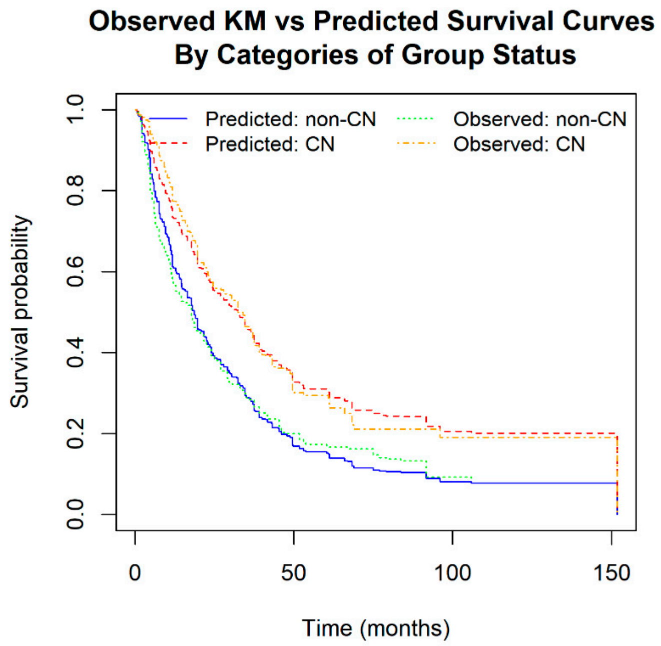

Supplement: Supplementary file 1 [file cancers-13-00695-s001.zip › Supplemental Data File 4.pdf]
