# Supplementary material for: Long-Term Survival Outcomes of Cytoreductive Nephrectomy Combined with Targeted Therapy for Metastatic Renal Cell Carcinoma: A Systematic Review and Individual Patient Data Meta-Analysis
Source: Cancers (Basel). 2021 Feb 9;13(4):695. doi: 10.3390/cancers13040695 (PMC7915816; doi:10.3390/cancers13040695)
Supplement: Supplementary file 1 [file cancers-13-00695-s001.zip › Supplemental Data File 5.pdf]

Supplemental Data File 5. Results of the two-stage survival meta-analyses.

<u>Forest plots</u>

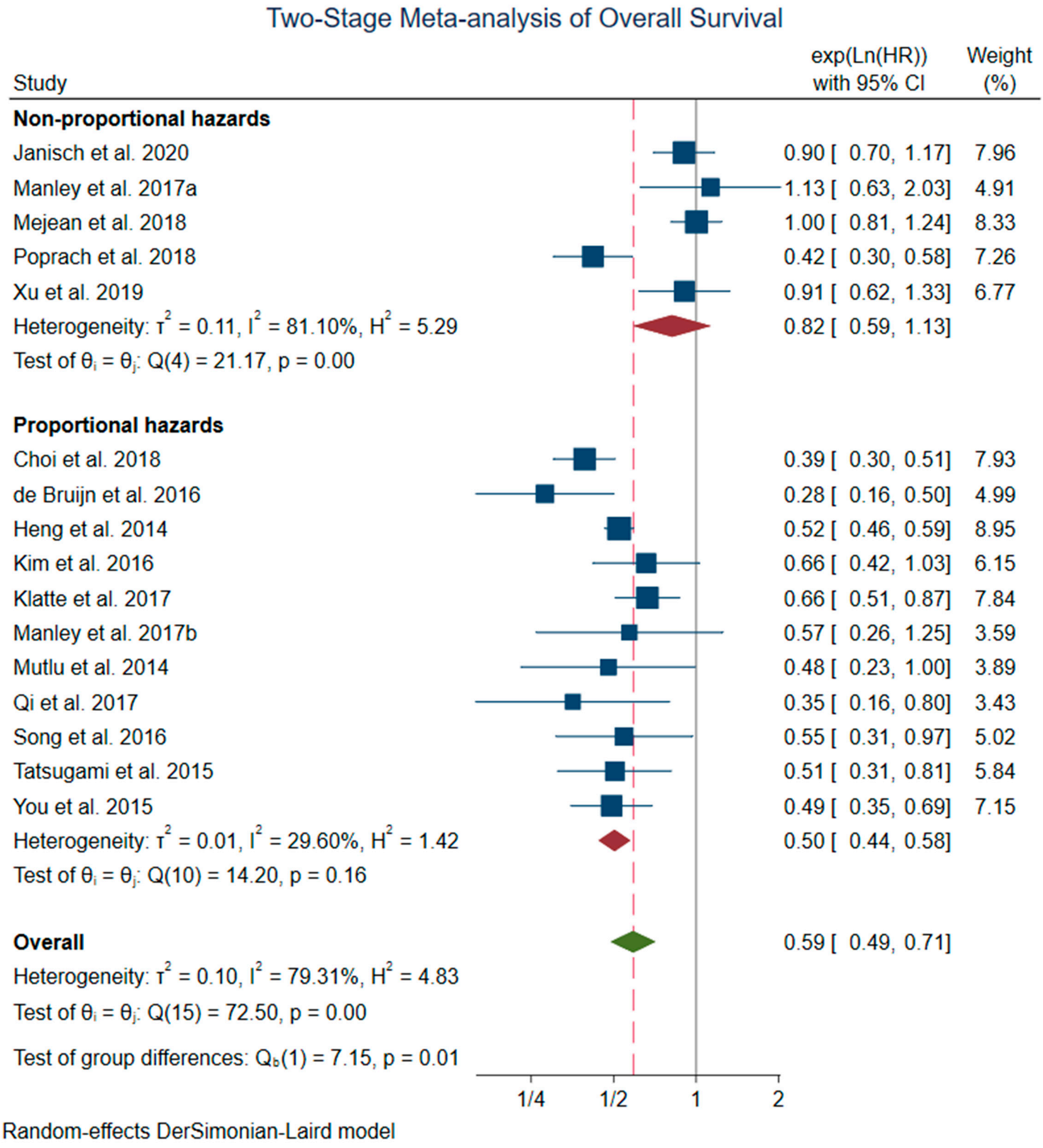

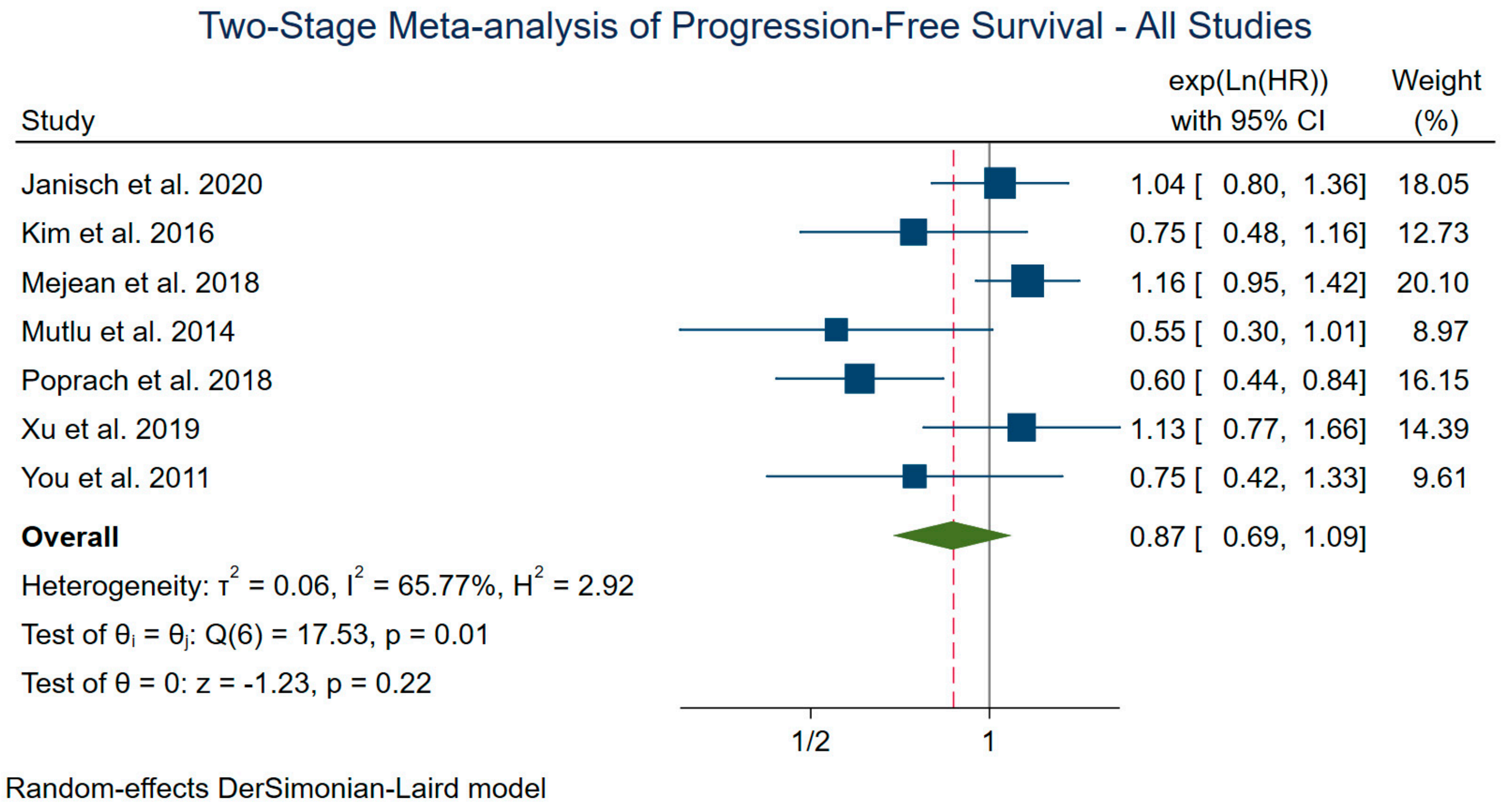
Two-Stage Meta-analysis of Progression-Free Survival - All Studies
Study
exp(Ln(HR)) with 95% CI
Weight (%)
Janisch et al. 2020 1.04 [0.80, 1.36] 18.05
Kim et al. 2016 0.75 [0.48, 1.16] 12.73
Mejean et al. 2018 1.16 [0.95, 1.42] 20.10
Mutlu et al. 2014 0.55 [0.30, 1.01] 8.97
Poprach et al. 2018 0.60 [0.44, 0.84] 16.15
Xu et al. 2019 1.13 [0.77, 1.66] 14.39
You et al. 2011 0.75 [0.42, 1.33] 9.61
Overall 0.87 [0.69, 1.09]
Heterogeneity: τ^2 = 0.06, I^2 = 65.77%, H^2 = 2.92
Test of $\theta_i = \theta_j$: Q(6) = 17.53, p = 0.01
Test of θ = 0: z = -1.23, p = 0.22
1/2
1
Random-effects DerSimonian-Laird model

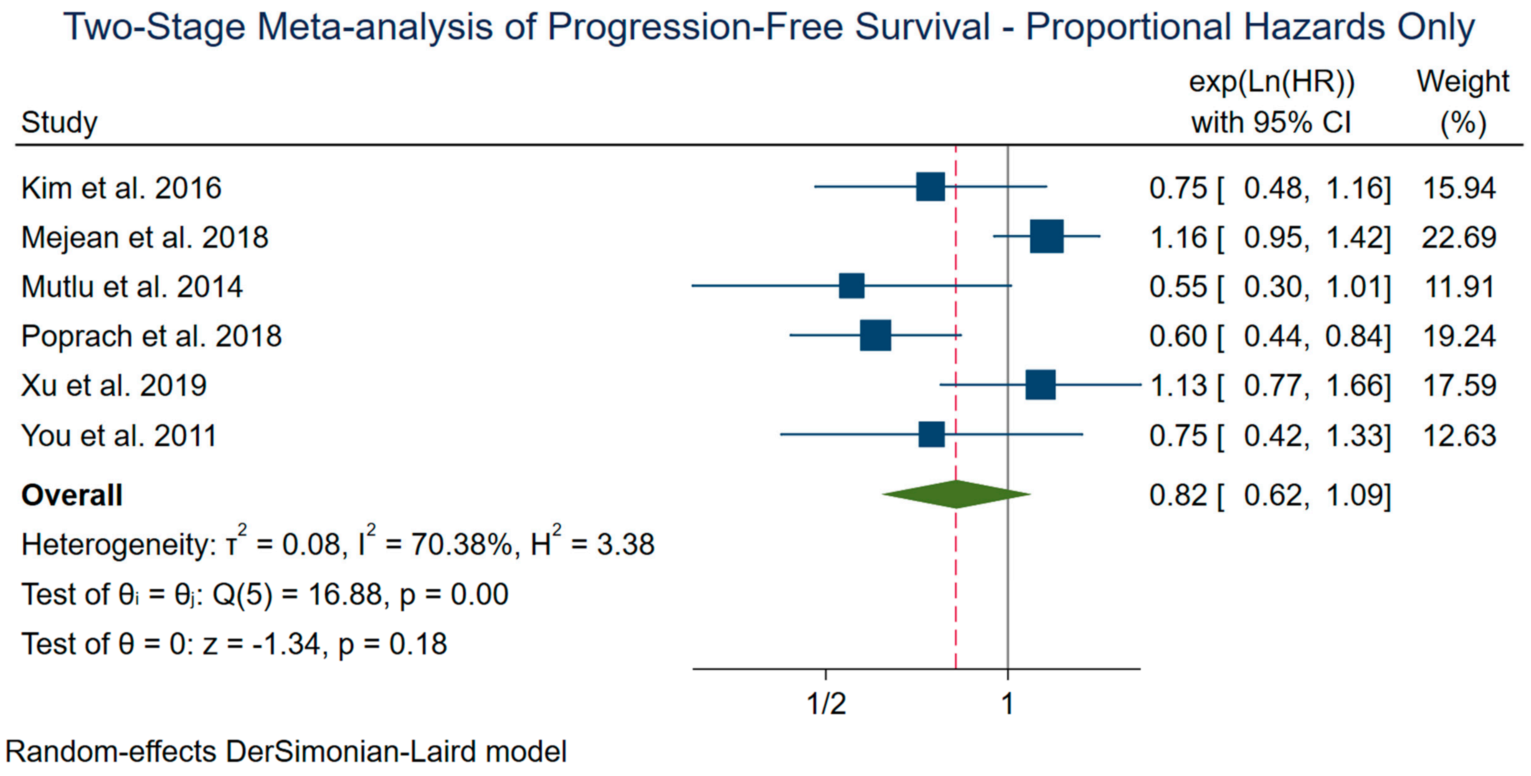
Two-Stage Meta-analysis of Progression-Free Survival - Proportional Hazards Only
Study
exp(Ln(HR)) with 95% CI
Weight (%)
Kim et al. 2016 0.75 [0.48, 1.16] 15.94
Mejean et al. 2018 1.16 [0.95, 1.42] 22.69
Mutlu et al. 2014 0.55 [0.30, 1.01] 11.91
Poprach et al. 2018 0.60 [0.44, 0.84] 19.24
Xu et al. 2019 1.13 [0.77, 1.66] 17.59
You et al. 2011 0.75 [0.42, 1.33] 12.63
Overall 0.82 [0.62, 1.09]
Heterogeneity: τ^2 = 0.08, I^2 = 70.38%, H^2 = 3.38
Test of $\theta_i = \theta_j$: Q(5) = 16.88, p = 0.00
Test of θ = 0: z = -1.34, p = 0.18
1/2
1
Random-effects DerSimonian-Laird model

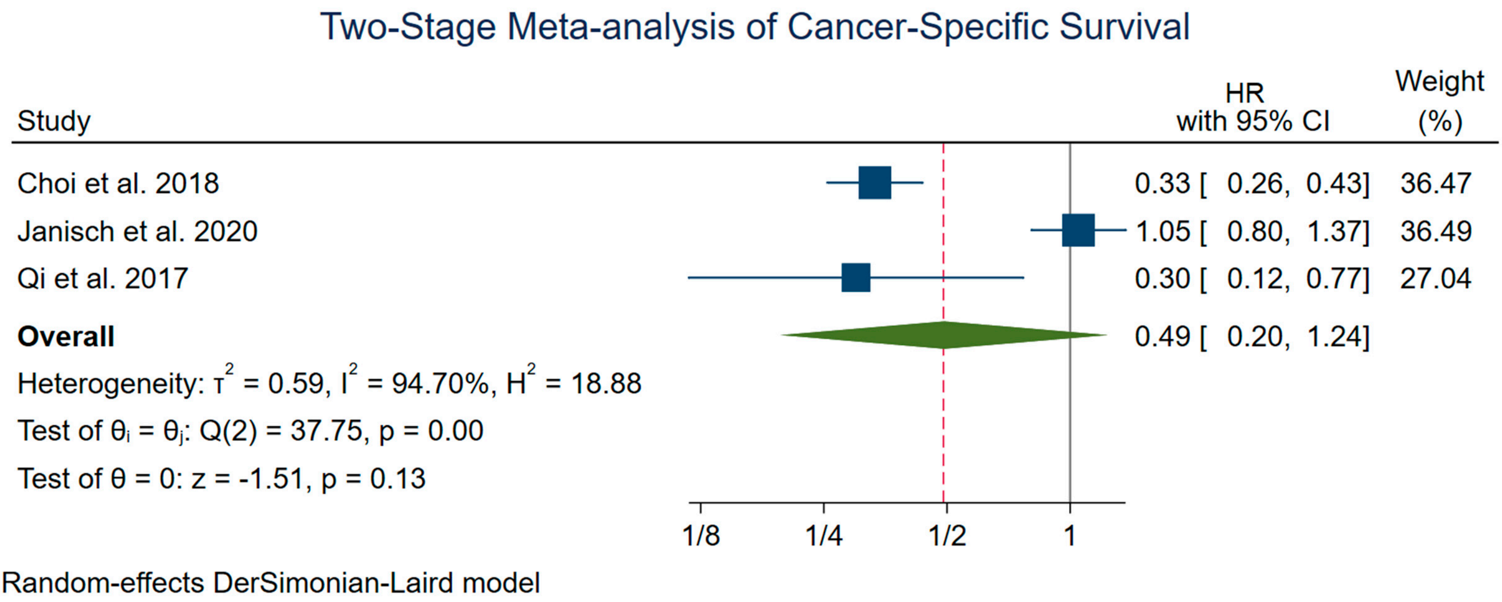

Funnel plots

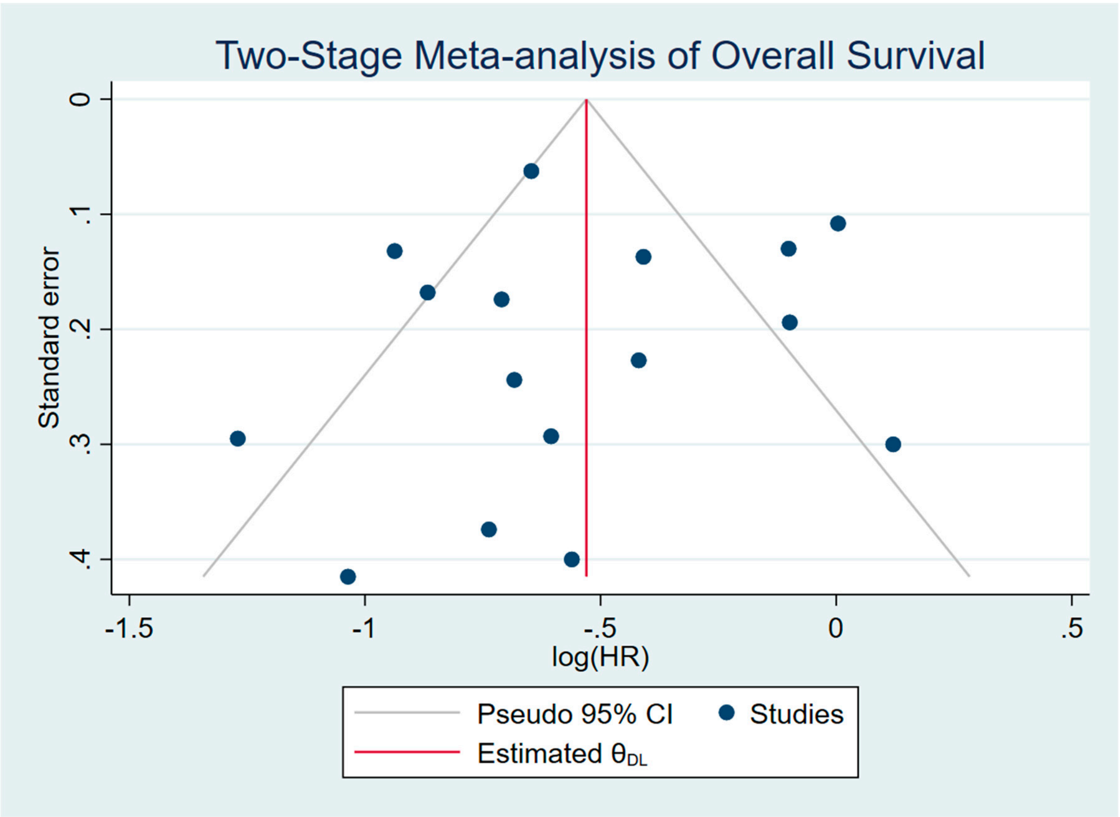

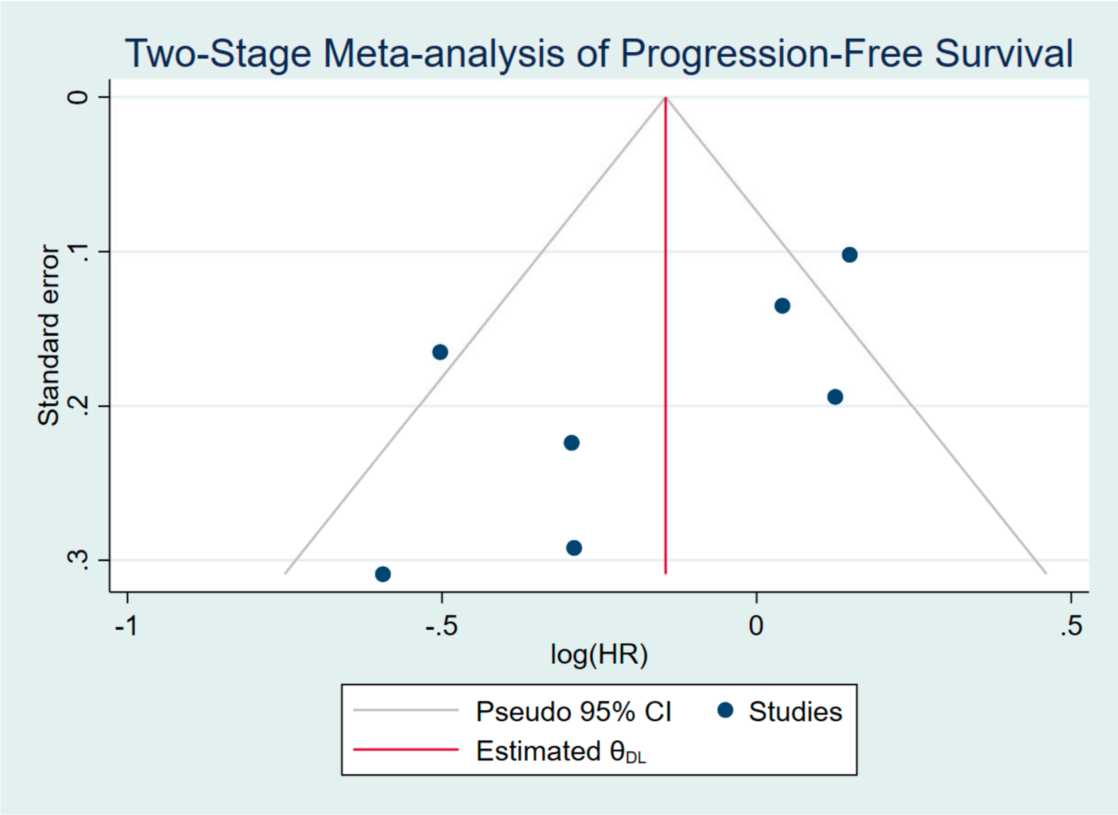
Two-Stage Meta-analysis of Progression-Free Survival
Standard error
0
.1
.2
.3
-1
-.5
0
.5
log(HR)
Pseudo 95% CI
Studies
Estimated θDL

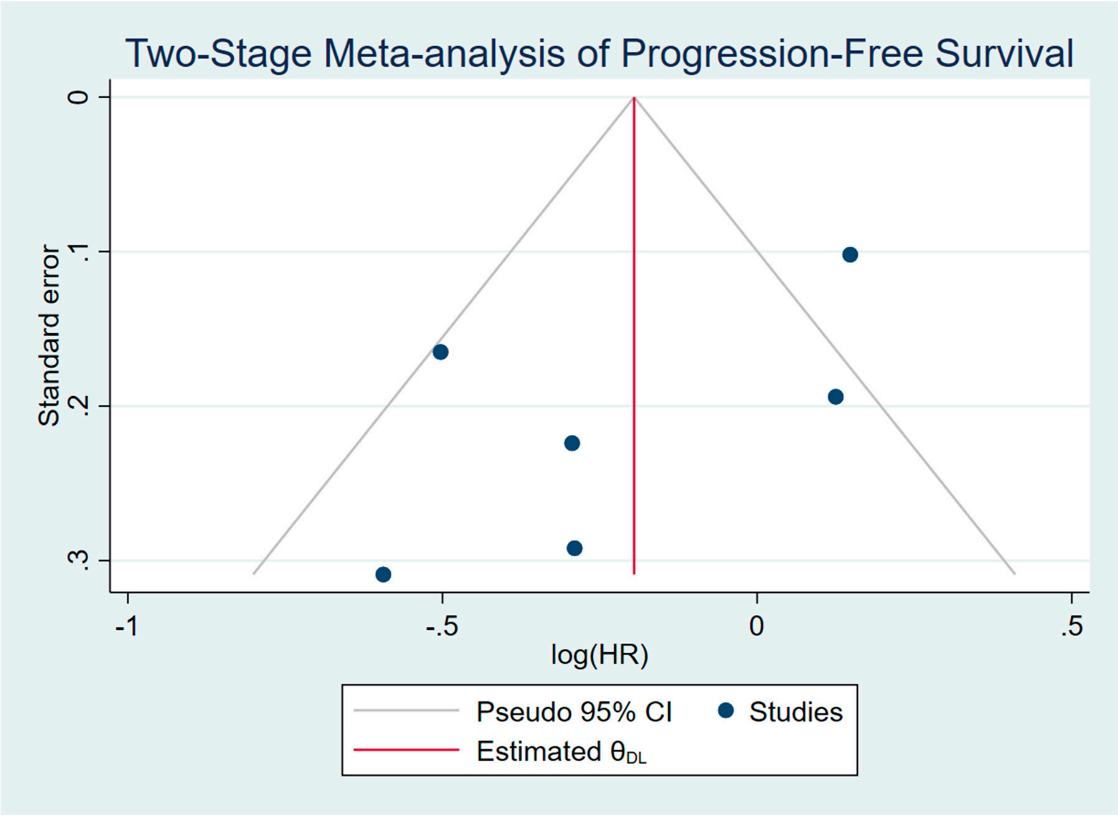
Two-Stage Meta-analysis of Progression-Free Survival
Standard error
0
.1
.2
.3
-1
-.5
0
.5
log(HR)
Pseudo 95% CI
Studies
Estimated θDL

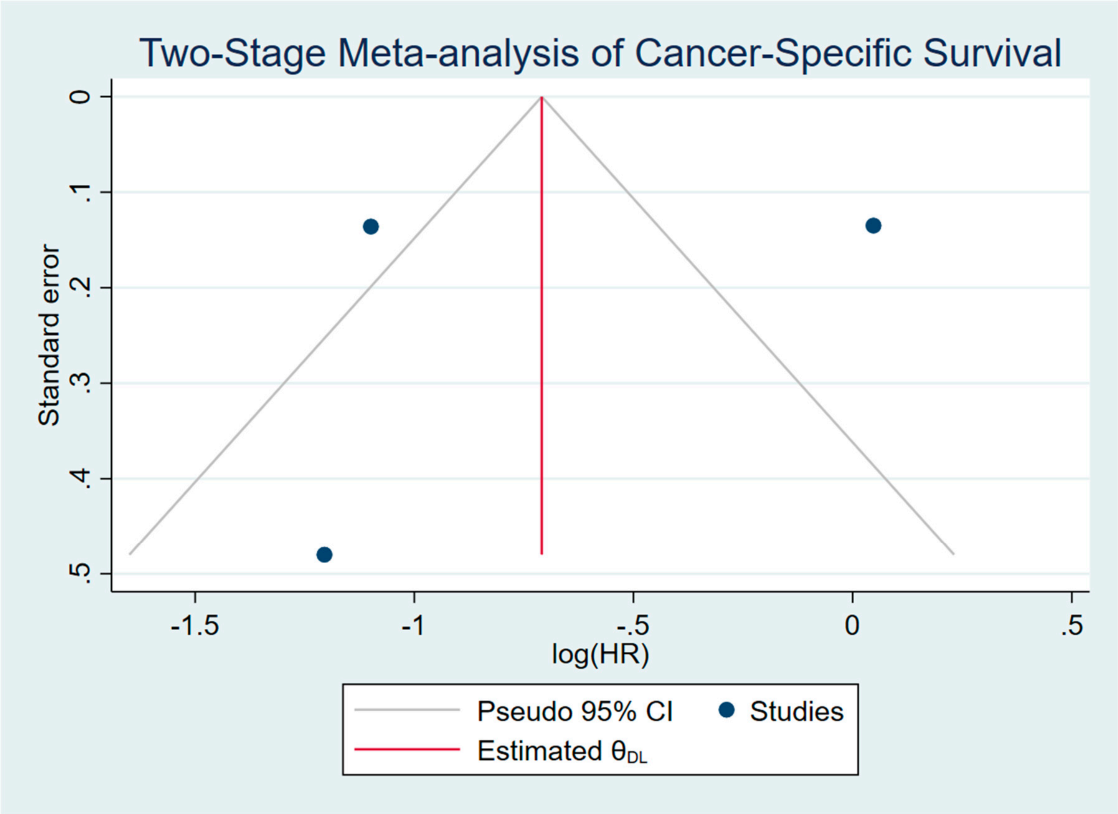
Two-Stage Meta-analysis of Cancer-Specific Survival
Standard error
0
.1
.2
.3
.4
.5
-1.5
-1
-.5
0
.5
log(HR)
Pseudo 95% CI
Studies
Estimated θDL
