# Supplementary material for: Long-Term Survival Outcomes of Cytoreductive Nephrectomy Combined with Targeted Therapy for Metastatic Renal Cell Carcinoma: A Systematic Review and Individual Patient Data Meta-Analysis
Source: Cancers (Basel). 2021 Feb 9;13(4):695. doi: 10.3390/cancers13040695 (PMC7915816; doi:10.3390/cancers13040695)
Supplement: Supplementary file 1 [file cancers-13-00695-s001.zip › Supplemental Data File 6.pdf]

Supplemental Data File 6. Results of the two-stage Bayesian meta-analyses.

Overall survival

	**Frequentist analysis**	**half-Normal prior-Informative Prior**	**Tibshirani prior-Uninformative prior**
Prior distribution μ	-	$N(0,10^6)$	$N(0,10^6)$
Pooled mean/posterior median	0.59	0.589	0.589
95% CI/CrI	0.49-0.71	0.476-0.723	0.476-0.724
Prior distribution τ	-	half-Normal (0,5)	$\sqrt{\sum_{i=1}^{k}\left(\frac{\tau}{\sigma_i^2+\tau^2}\right)^2}$
Estimated/posterior median	0.10	0.333	0.332
95% CI/CrI	-	0.186-0.525	0.183-0.529

CI: confidence interval; CrI: credibility interval

Progression-Free Survival

	**Frequentist analysis**	**half-Normal prior-Informative Prior**	**Tibshirani prior-Uninformative prior**
Prior distribution μ	-	$N(0,10^6)$	$N(0,10^6)$
Pooled/posterior median	0.87	0.871	0.870
95% CI/CrI	0.69-1.09	0.644-1.138	0.640-1.142
Prior distribution τ	-	half-Normal (0,5)	$\sqrt{\sum_{i=1}^{k}\left(\frac{\tau}{\sigma_i^2+\tau^2}\right)^2}$
Estimated/posterior median	0.06	0.264	0.264
95% CI/CrI	-	0.049-0.564	0.058-0.577

CI: confidence interval; CrI: credibility interval

Cancer-Specific Survival

	**Frequentist analysis**	**half-Normal prior-Informative Prior**	**Tibshirani prior-Uninformative prior**
Prior distribution μ	-	$N(0,10^6)$	$N(0,10^6)$
Pooled/posterior median	0.49	0.509	0.502
95% CI/CrI	0.20-1.24	0.214-1.156	0.079-2.910
Prior distribution τ	-	half-Normal (0,5)	$\sqrt{\sum_{i=1}^{k}\left(\frac{\tau}{\sigma_i^2+\tau^2}\right)^2}$
Estimated/posterior median	0.59	0.590	0.833
95% CI/CrI	-	0.242-1.093	0.188-3.207

CI: confidence interval; CrI: credibility interval

Forest plot of two-step Bayesian meta-analysis for overall survival using an informative prior

Two-step Bayesian Meta-analysis of Overall Survival

Study	Hazard Ratio	95% CI
Choi et al. 2018	0.392	[0.302, 0.507]
de Bruijn et al. 2016	0.281	[0.158, 0.501]
Heng et al. 2014	0.524	[0.463, 0.592]
Janisch et al. 2020	0.904	[0.701, 1.166]
Kim et al. 2016	0.658	[0.422, 1.026]
Klatte et al. 2017	0.664	[0.508, 0.869]
Manley et al. 2017	1.129	[0.627, 2.032]
Mejean et al. 2018	1.004	[0.812, 1.241]
Mutlu et al. 2014	0.479	[0.230, 0.996]
Poprach et al. 2018	0.420	[0.302, 0.584]
Qi et al. 2017	0.355	[0.157, 0.800]
Song et al. 2016	0.546	[0.308, 0.970]
Tatsugami et al. 2015	0.505	[0.313, 0.815]
Xu et al. 2019	0.906	[0.620, 1.325]
You et al. 2015	0.492	[0.350, 0.691]
Manley_b et al. 2017	0.571	[0.261, 1.250]
**Mean**	**0.589**	**[0.476, 0.723]**
**Prediction**	**0.589**	**[0.278, 1.236]**

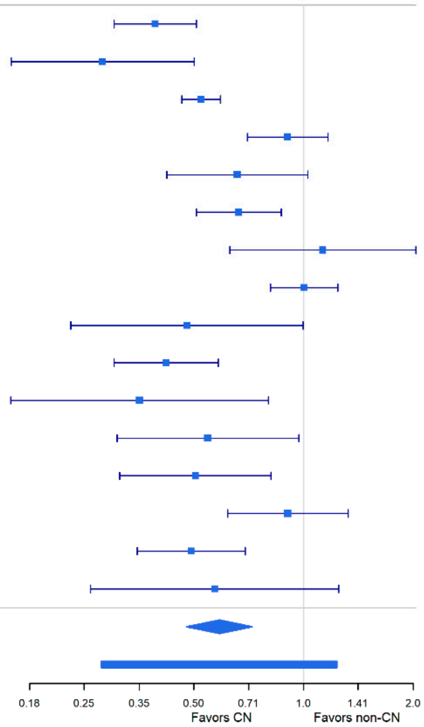

Heterogeneity (tau): 0.33 [0.19, 0.53]

Forest plot of two-step Bayesian meta-analysis for overall survival using an uninformative prior

Two-step Bayesian Meta-analysis of Overall Survival

Study	Hazard Ratio	95% CI
Choi et al. 2018	0.392	[0.302, 0.507]
de Bruijn et al. 2016	0.281	[0.158, 0.501]
Heng et al. 2014	0.524	[0.463, 0.592]
Janisch et al. 2020	0.904	[0.701, 1.166]
Kim et al. 2016	0.658	[0.422, 1.026]
Klatte et al. 2017	0.664	[0.508, 0.869]
Manley et al. 2017	1.129	[0.627, 2.032]
Mejean et al. 2018	1.004	[0.812, 1.241]
Mutlu et al. 2014	0.479	[0.230, 0.996]
Poprach et al. 2018	0.420	[0.302, 0.584]
Qi et al. 2017	0.355	[0.157, 0.800]
Song et al. 2016	0.546	[0.308, 0.970]
Tatsugami et al. 2015	0.505	[0.313, 0.815]
Xu et al. 2019	0.906	[0.620, 1.325]
You et al. 2015	0.492	[0.350, 0.691]
Manley_b et al. 2017	0.571	[0.261, 1.250]
**Mean**	**0.589**	**[0.476, 0.724]**
**Prediction**	**0.589**	**[0.278, 1.237]**

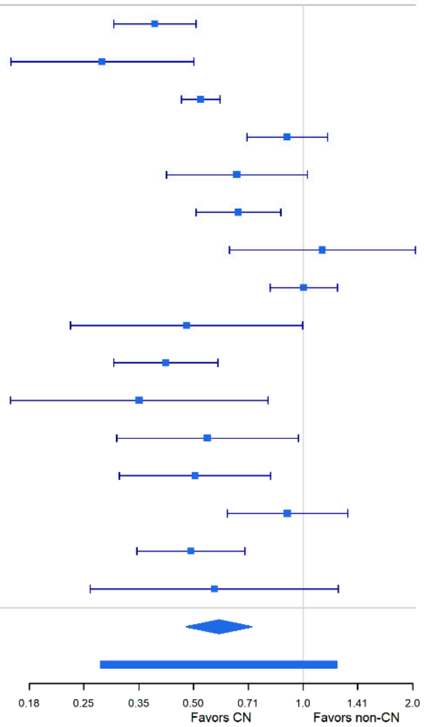

Heterogeneity (tau): 0.33 [0.18, 0.53]

Forest plot of two-step Bayesian meta-analysis for progression-free survival using an informative prior

Two-step Bayesian Meta-analysis of Progression-Free Survival

Study	Hazard Ratio	95% CI
Janisch et al. 2020	1.042	[0.800, 1.357]
Kim et al. 2016	0.745	[0.480, 1.156]
Mejean et al. 2018	1.160	[0.949, 1.416]
Mutlu et al. 2014	0.552	[0.301, 1.012]
Poprach et al. 2018	0.605	[0.438, 0.836]
Xu et al. 2019	1.133	[0.775, 1.657]
You et al. 2011	0.748	[0.422, 1.326]
**Mean**	**0.871**	**[0.644, 1.138]**
**Prediction**	**0.876**	**[0.418, 1.735]**

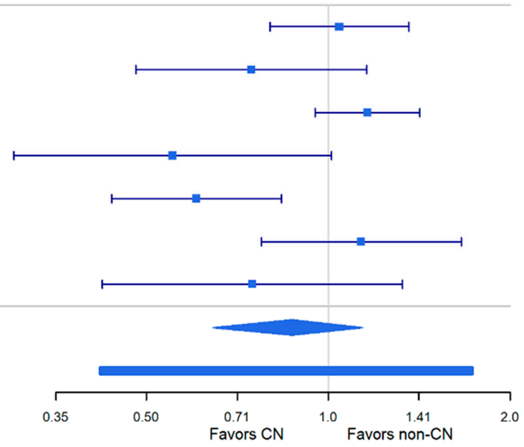

Heterogeneity (tau): 0.264 [0.049, 0.564]

Forest plot of two-step Bayesian meta-analysis for progression-free survival using an uninformative prior

Two-step Bayesian Meta-analysis of Progression-Free Survival

Study	Hazard Ratio	95% CI
Janisch et al. 2020	1.042	[0.800, 1.357]
Kim et al. 2016	0.745	[0.480, 1.156]
Mejean et al. 2018	1.160	[0.949, 1.416]
Mutlu et al. 2014	0.552	[0.301, 1.012]
Poprach et al. 2018	0.605	[0.438, 0.836]
Xu et al. 2019	1.133	[0.775, 1.657]
You et al. 2011	0.748	[0.422, 1.326]
**Mean**	**0.870**	**[0.640, 1.142]**
**Prediction**	**0.874**	**[0.412, 1.759]**

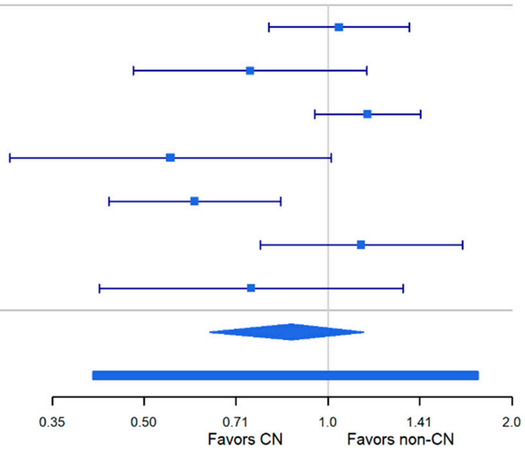

Heterogeneity (tau): 0.264 [0.058, 0.577]

Forest plot of two-step Bayesian meta-analysis for cancer-specific survival using an informative prior

Two-step Bayesian Meta-analysis of Cancer-Specific Survival

Study	Hazard Ratio	95% CI
Choi et al. 2018	0.333	[0.255, 0.435]
Janisch et al. 2020	1.049	[0.805, 1.366]
Qi et al. 2017	0.300	[0.117, 0.768]
**Mean**	**0.509**	**[0.214, 1.156]**
**Prediction**	**0.509**	**[0.099, 2.486]**

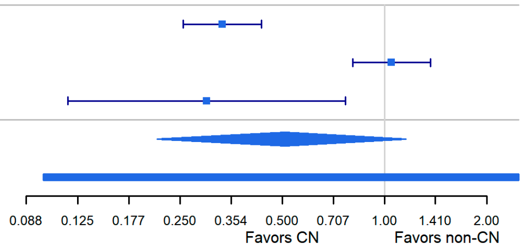

Heterogeneity (tau): 0.59 [0.24, 1.09]

Forest plot of two-step Bayesian meta-analysis for cancer-specific survival using an uninformative prior

Two-step Bayesian Meta-analysis of Cancer-Specific Survival

Study	Hazard Ratio	95% CI
Choi et al. 2018	0.333	[0.255, 0.435]
Janisch et al. 2020	1.049	[0.805, 1.366]
Qi et al. 2017	0.300	[0.117, 0.768]
**Mean**	**0.502**	**[0.079, 2.910]**
**Prediction**	**0.503**	**[0.013, 17.152]**

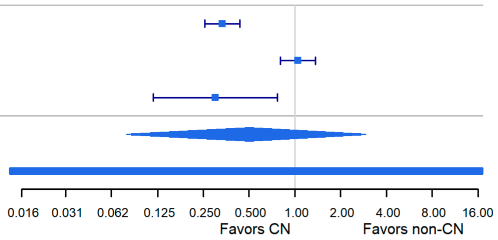

Heterogeneity (tau): 0.83 [0.19, 3.21]
