# Supplementary material for: Long-Term Survival Outcomes of Cytoreductive Nephrectomy Combined with Targeted Therapy for Metastatic Renal Cell Carcinoma: A Systematic Review and Individual Patient Data Meta-Analysis
Source: Cancers (Basel). 2021 Feb 9;13(4):695. doi: 10.3390/cancers13040695 (PMC7915816; doi:10.3390/cancers13040695)
Supplement: Supplementary file 1 [file cancers-13-00695-s001.zip › Supplemental Data File 7.pdf]

Supplemental Data File 7. Subgroup analysis of upfront CN for overall, progression-free, and cancer-specific survival estimates using reconstructed survival information.

One-stage Meta-Analysis		Overall Survival		Progression-Free Survival		Cancer-Specific Survival	
		**Relative Effect of CN Versus non-CN (95% CI/CrI)**	***P*-value for Relative Effect**	**Relative Effect of CN Versus non-CN (95% CI/CrI)**	***P*-value for Relative Effect**	**Relative Effect of CN Versus non-CN (95% CI/CrI)**	***P*-value for Relative Effect**
Frequentist Approach	Cox Proportional Hazards Model	0.70[a] (0.63-0.78)	< 0.0001	0.94[b] (0.59-1.07)	0.328	0.97[c] (0.75-1.25)	0.814
	Life Expectancy Difference (up to 3 years)	4.0 months (2.9-5.2)	< 0.0001	0.6 months [(-0.8)-(1.9)]	0.389	3.4 months (0.4-6.5)	0.029
	Life Expectancy Ratio (up to 3 years)	1.23 (1.16-1.31)	< 0.0001	1.05 (0.94-1.16)	0.390	1.16 (1.02-1.33)	0.030
	Life Expectancy Difference (up to 5 years)	6.4 months (4.6-8.2)	< 0.0001	0.7 months [(-1.3)-(2.8)]	0.476	3.4 months [(-1.7)-(8.5)]	0.192
	Life Expectancy Ratio (up to 5 years)	1.31 (1.21-1.41)	< 0.0001	1.05 (0.94-1.03)	0.476	1.12 (0.95-1.33)	0.192
Bayesian Approach	Cox Proportional Hazards Model	0.59 (0.55-0.63)	N/A	0.94 (0.84-1.07)	N/A	0.98 (0.75-1.23)	N/A

CN: Cytoreductive nephrectomy; CI: confidence interval; CrI: credibility interval; N/A: not available.
[a]Assessing for non-proportional hazards *P*-value = 0.290, from the Grambsch-Therneau test
[b]Assessing for non-proportional hazards *P*-value = 0.500, from the Grambsch-Therneau test
[c]Assessing for non-proportional hazards *P*-value = 0.0058, from the Grambsch-Therneau test
